# Scalable synthesis of polyhydroxy iron-modified kaolin *via* low-temperature hydrolysis-polymerization for efficient adsorption of direct black 38

**DOI:** 10.1039/d5ra05883j

**Published:** 2025-11-24

**Authors:** Xi Duo, Qingyi Qian, Yunsong Zhang, Liangliang Bao, Qier Mu, Hui Yan, Latai Ga, Zhimei Zhong

**Affiliations:** a College of Science, Inner Mongolia Agricultural University Hohhot 010018 Inner Mongolia China yanhui@imau.edu.cn zhimeihappy@126.com; b Inner Mongolia Key Laboratory of Soil Quality and Nutrient Resource Hohhot 010018 Inner Mongolia China; c College of Animal Science, Inner Mongolia Agricultural University Hohhot 010018 Inner Mongolia China; d College of Materials Science and Art Design, Inner Mongolia Agricultural University Hohhot 010018 Inner Mongolia China; e School of Renewable Energy, Inner Mongolia University of Technology Ordos 017010 Inner Mongolia China galtai@imut.edu.cn

## Abstract

Kaolin (KC) has highly promising prospects in the purification of the water environment and treatment of organic pollutants. However, its low adsorption capacity, poor selectivity and low reusability significantly constrain its further application as an agent for treating water pollution. Herein, a novel kaolin adsorbent, namely polyhydroxy iron-modified kaolin (PIK), has been devised through low-temperature liquid-phase synthesis and impregnation approaches, surmounting the limitations of non-selectivity and low adsorption capacity. The adsorbent accomplished selective adsorption of Direct Black 38 (DB38) with an adsorption capacity of 76.1 mg g^−1^ and could maintain an adsorption efficiency of 82.0% even after being reused three times. This work demonstrates the remarkable adsorption performance of PIK and provides a promising strategy for the large-scale preparation and practical application of high-capacity kaolin adsorbents at low temperatures.

## Abstract 2

The adsorption mechanism of PIK for DB38 dyes (a), zeta potential (b), distribution of iron ion forms (c) and comparison of DB38 adsorption effects between KC and PIK (d).

## Graphical abstract

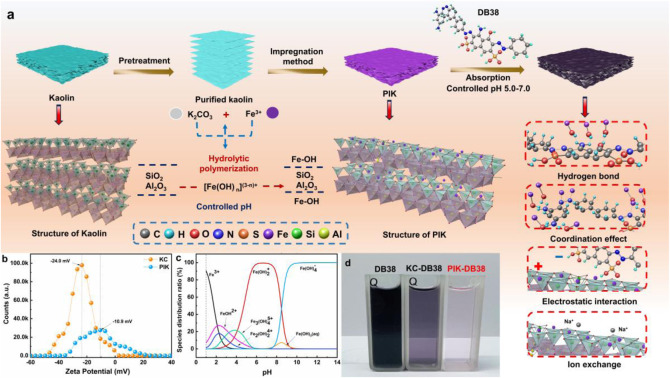

## 1. Introduction

With the continuous advancement of the light industry, diverse types of dyes have been synthesized and widely utilized in various production procedures, causing a large amount of synthetic dye wastewater to be discharged into rivers and lakes, resulting in pollution to varying degrees of the aquatic environment.^1,2^ Synthetic dye wastewater has characteristics such as intense coloration, resistance to degradation, high content of toxic substances, complex composition, and strong acidity or alkalinity.^3^ These factors can give rise to the eutrophication of water bodies, excessive consumption of dissolved oxygen, pose a threat to the survival of aquatic animals and plants, and affect human health through the transmission of the ecological chain.^4^ Therefore, the development of practical and effective strategies for mitigating dyes contamination in aquatic ecosystems has become an urgent issue that requires prompt attention and intervention.

Currently, there are three principal methods for eliminating synthetic dyes from water environments: physical, chemical, and biological methods.^5^ The adsorption method is widely adopted because of its advantages like strong operability, good economic feasibility, environmental friendliness, low cost, wide applicability and high treatment efficiency.^6^ Natural materials, including biochar, sepiolite and kaolinite clay minerals, as well as novel materials such as metal–organic frameworks (MOFs), exhibit efficient adsorption and immobilization capabilities toward organic dye pollutants.^7–10^ However, the potential toxicity and high preparation cost of adsorbents like activated carbon limit their practical application scope.^11^ Kaolin is a natural clay mineral that can be directly obtained from nature.^12–14^ Due to its stable structure, low cost and abundant reserves, it is widely utilized in fields such as ceramics, absorbents, coatings, catalyst carriers, molecular sieve preparation and environmental remediation.^15,16^ Nevertheless, the inherent adsorption capacity of raw kaolinite is limited. To enhance its adsorption efficiency for specific contaminants, researchers have utilized kaolinite as a matrix. Through modification strategies such as enlarging the interlayer spacing,^17,18^ increasing the specific surface area,^19–21^ enriching the variety of functional groups,^22,23^ or modulating surface hydrophilicity.^24,25^ Kaolinite-based composite materials exhibiting both high adsorption capacity and reusability have been successfully fabricated, significantly expanding their application potential. Among these strategies, modification using metal elements represents a common approach. Adsorptive or catalytic active centers are typically formed on the kaolinite surface *via* impregnation, deposition, or reduction methods. These centers function synergistically with the kaolinite substrate to degrade or adsorb contaminants^26,27^^.^

Iron is a non-toxic transition metal element. Its ions (Fe^3+^) possess a unique electronic configuration, hydrated ionic radius, and stable physicochemical properties. Numerous studies have demonstrated that iron ion-modified kaolinite composites exhibit excellent adsorption capacities for diverse pollutants, including antibiotics,^26,27^ organic dyes,^28,29^ endocrine-disrupting compounds (EDCs),^30,31^ and heavy metal ions.^32,33^ Despite the favorable performance of iron-modified kaolinite in dye removal, large-scale production is hampered by factors such as low iron ion loading efficiency and high energy consumption during preparation, thereby limiting its application in pollution control. Furthermore, the stability of adsorbents modified with iron ions varies considerably. Although K. Győrfi *et al.* increased iron ion loading by enhancing kaolinite porosity,^34^ this approach led to significantly increased costs and caused environmental pollution due to waste acid generation. Current research confirms that certain transition metal elements, including Fe^3+^/Fe^2+^ and Cu^2+^, are prone to hydrolytic polymerization transformations across various pH environments.^35,36^ Iron ions can maintain stability in solution through the formation of coordination bonds with water molecules and OH^−^ ions.^37,38^ When the environmental pH changes, the configuration and polymerization state of hydrated Fe^3+^/Fe^2+^ ions are altered, and the charge characteristics of the resulting polymeric species are correspondingly modified.^35,39^ Utilizing this characteristic, polymeric ions of transition metals can be formed, which are capable of binding to organic pollutant molecules *via* hydrogen bonding and electrostatic interactions. Adsorbents prepared using this method exhibit significantly enhanced adsorption capacities for organic pollutants compared to those modified by conventional iron ion methods.^40,41^ To improve the utilization efficiency of such polymeric ions, they can be immobilized and stabilized on kaolinite surfaces possessing porous structures and substantial surface area. Concurrently, the enrichment of polyhydroxy iron ions on the kaolinite surface increases the number of hydroxyl functional groups, creating numerous adsorption sites for pollutant removal by kaolinite-based adsorbents. J. Wan *et al.* employed a kaolinite/lanthanum carbonate composite to simultaneously adsorb orthophosphate (PO_4_^3−^) and 1-hydroxyethylidene-1,1-diphosphonic acid (HEDP), finding that carbonate ions inhibited adsorption performance.^42^ Potassium carbonate (K_2_CO_3_), serving as a multifunctional modifying agent, can maintain soil–water pH balance and promote metal ion hydrolysis, demonstrating its potential as an auxiliary reagent for the hydrolysis of transition metal ions.

**Fig. 1:**
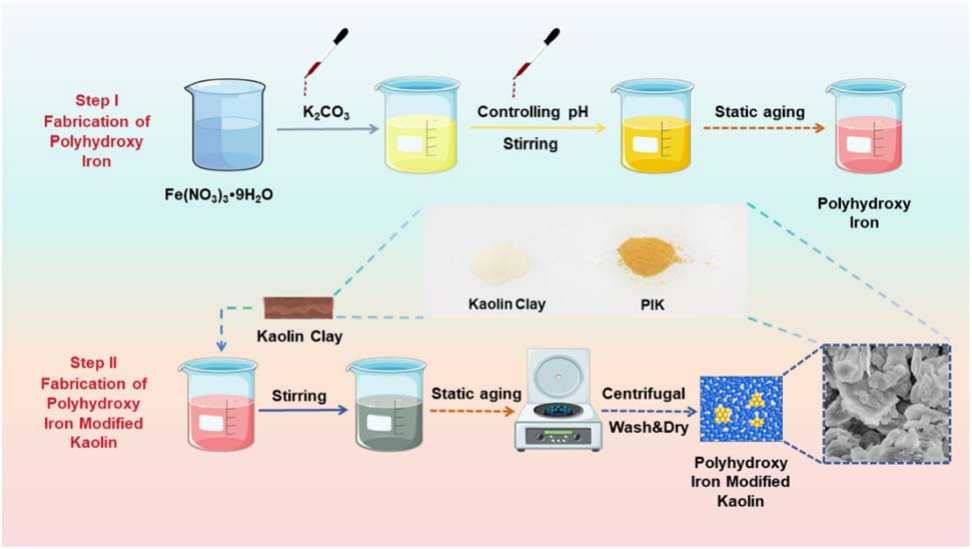
The schematic diagram for the preparation of kaolin modified with polyhydroxy iron.

This study is based on previous research and uses K_2_CO_3_ and Fe(NO_3_)_3_ as raw materials to prepare polyhydroxy iron precursor (PI). K_2_CO_3_ is used to improve the hydrolysis degree and stability of iron ions, making it a colloid carrying positive charges. The adsorbent PIK was prepared by loading polyhydroxy iron on the surface of native kaolin. The preparation process is shown in Fig. 1. At the same time as increasing the specific surface area and porosity of kaolin, increasing the active adsorption sites on the surface of kaolin, rationalizing the distribution of iron ions on kaolin, fundamentally improving the loading rate of iron ions on kaolin surface, maintaining the balance between kaolin pore structure and effective loaded iron ions, thereby improving the adsorption efficiency of iron ion modified kaolin for organic pollutants. Using PIK to remove DB38 from water as a model reaction, the influence of PIK stability on iron ion loading in kaolin, the adsorption performance of PIK adsorbent on DB38 before and after modification, and the adsorption mechanism of PIK on DB38 were studied in detail, providing reference for the resource utilization of kaolin.

## 2. Results and discussion

### 2.1. Characterization of surface properties and morphologies

**Fig. 2:**
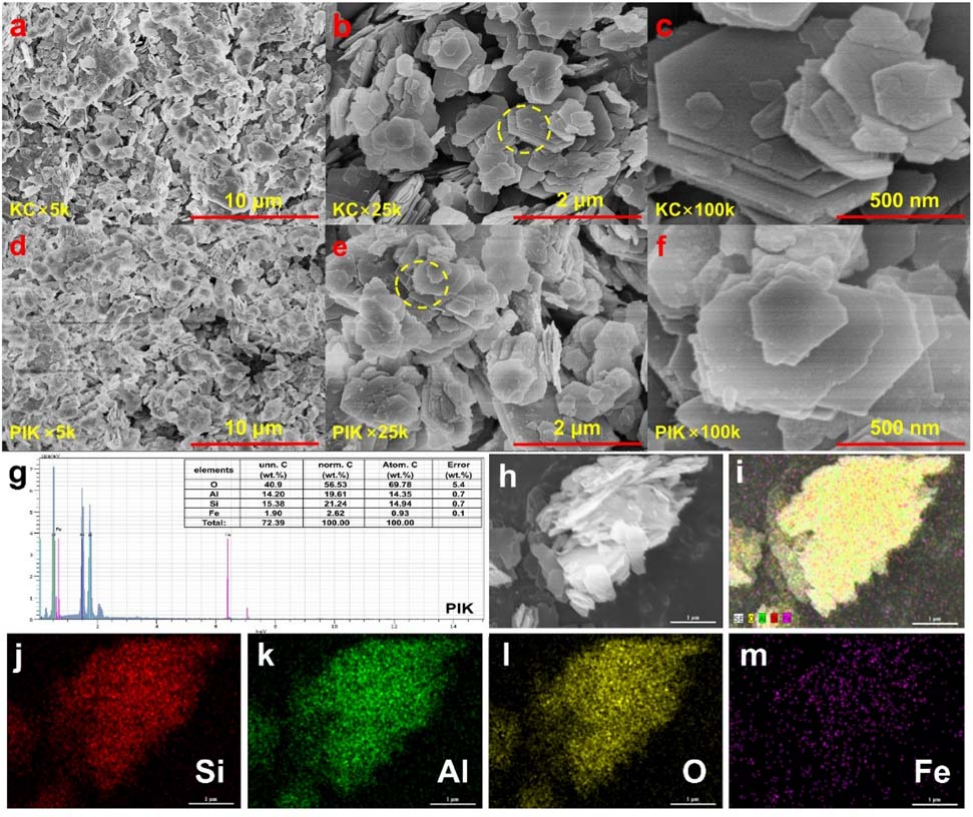
The SEM images of KC and PIK (a–f) and the EDS of PIK (g–m).

The scanning electron microscopy (SEM) showed that in contrast to KC with a sheet-like stacked structure (Fig. 2a–c and S1), the modification of polyhydroxy iron had a certain influence on the morphology of kaolin, and partial edge corrosion and passivation phenomena emerged on the surface of KC (Fig. 2d–f). The OH^−^ generated subsequent to the hydrolysis of K_2_CO_3_ would corrode the silica layer in the kaolin structure, exposing a greater number of pores and resulting in a reduction in the average pore size of PIK. Although the loading of PI on the surface of kaolin clay diminished its pore volume, the 22.49 m^2^ g^−1^ specific surface area of PIK is still 1.58 times that of KC (Table S1).

The calculation outcomes of EDS elemental distribution disclose that the elemental distribution on the surface of KC is characterized by a substantial proportion of Si, Al, and O (Fig. S2 and S3). It can be seen from Fig. 2g that compared with KC, the content of Si and O in PIK decreased, while the content of Al relatively increased. This is due to the modification of polyhydroxy iron, where some silicon elements in the silicon-oxygen tetrahedron in the kaolin structure are removed. Simultaneously, iron elements are not detected on surface KC (Fig. S4), which may be attributed to the scant iron content in KC. As show in Fig. 2h–m, combining EDS mapping and characterization of individual elements, the various elements on the surface of PIK exhibit a uniform distribution state. The iron relative content of 2.62% in PIK suggests that Fe element can permeate the interlayer or surface structure of kaolin through ion exchange or electrostatic interaction (Fig. 2g).

**Fig. 3:**
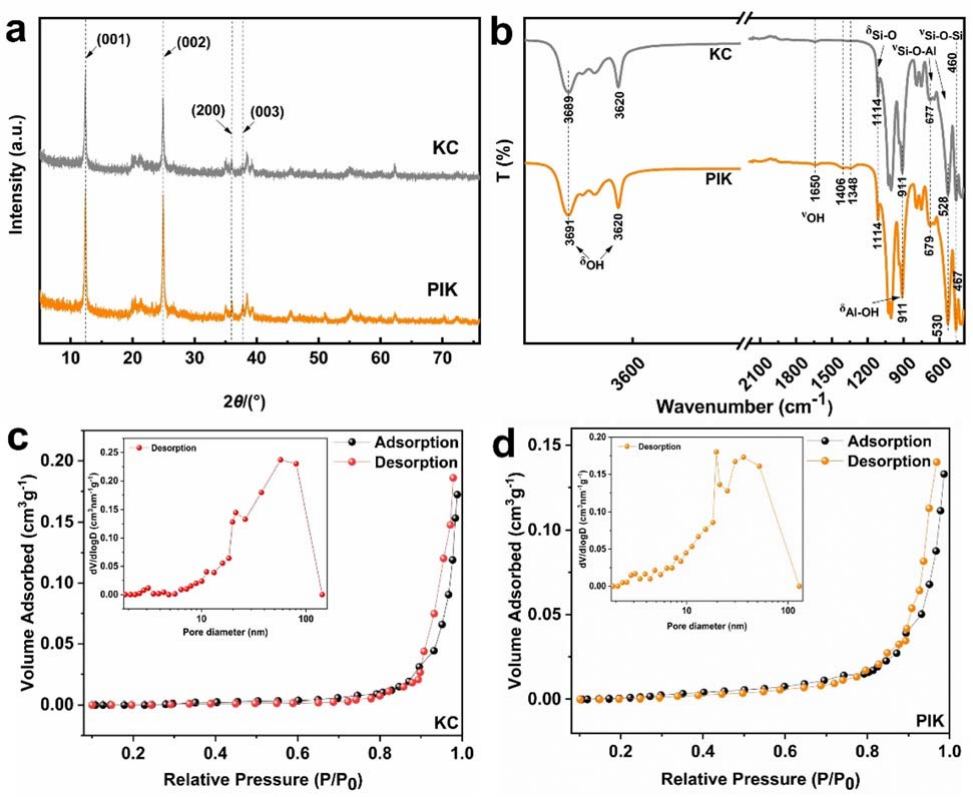
The XRD patterns (a), FT-IR(b), nitrogen adsorption–desorption isotherms(c), pore diameter distribution (d) of KC and PIK.

The XRD results of KC and PIK are shown in Fig. 3a. The main components in KC and PIK are kaolin, and the diffraction peak (2*θ*) is predominantly concentrated within the range of 11° to 40°. The characteristic diffraction peaks of kaolin appear at 12.37°, 24.96°, 36.08°, and 38.56°, corresponding respectively to the (001), (002), (200), and (003) crystal planes of kaolin (JCPDS No. 79-1570). It is notable that no new diffraction peaks emerged for the PIK at 2*θ* of 14.2° (γ-FeOOH), 21.2° (α-FeOOH), 26.7° (β-FeOOH), 33.2° (Fe_2_O_3_) or 35.4° (Fe_3_O_4_), indicating the absence of iron oxides or hydroxides in the PIK.^43–45^ The sharp diffraction peaks indicate that both KC and PIK possess high crystallinity, and the crystal structure has not been destroyed. Concurrently, in compared with the diffraction peaks of KC, the intensity of PIK diffraction peaks at 12.37° and 24.92° is marginally higher, without significant diffraction peak shift. Nevertheless, the diffraction peak at 36.2° corresponding to the crystal plane (200) is slightly shifted, indicating that Fe^3+^ (0.78 Å) has not fully entered the kaolin structure to substitute for aluminum or silicon ions, and a small quantity of Fe^3+^ ions have undergone eutectic substitution in the structure.^46^ This result is consistent with the test results of Zhu *et al.*^47^ The modification process had a certain degree of impact on the structure of KC, suggesting that Fe element exists on the surface of PIK in an amorphous form.

The FT-IR measurements were performed on KC and PIK within the range of 100 cm^−1^ to 4000 cm^−1^, and the results are depicted in Fig. 3b. Sharp absorption peaks within the range of 3689 cm^−1^ to 3620 cm^−1^ are observed in both KC and PIK, which are attributed to the stretching vibrations of –OH from adsorbed water.^48^ Absorption peaks at 1114 cm^−1^, 1027 cm^−1^, and 911 cm^−1^ in KC and PIK correspond to the asymmetric Si–O and Al–O stretching vibrations within the aluminosilicate structure. In contrast to KC, PIK manifests an augmented number of surface hydroxyl groups. The weak absorption peak at 1406 cm^−1^ is attributed to the adsorption of CO_3_^2−^ on the surface of PIK.^49^ Furthermore, a new absorption band (1348–1402 cm^−1^) has emerged in PIK, which is the stretching vibration absorption peak of NO_3_^−^.^50^ The bending vibration peaks of Si–O bonds in KC at 677 cm^−1^ and 528 cm^−1^ exhibit a redshift phenomenon, which may be due to the formation of Si–O–Fe bonds during the modification with polyhydroxy iron, inducing the absorption peaks to shift towards longer wavelengths.^20^ In the fingerprint region, no new absorption peak corresponding to the Fe–O bond was detected near 470 cm^−1^ and 560 cm^−1^,^51,52^ which may be due to the low content of iron elements or overlapping with other characteristic absorption peaks, thereby rendering it challenging to be identified or remain undetected.

Fig. 3c and d show the N_2_ adsorption–desorption isotherms and BET analysis of KC and PIK, respectively. The results showed that the specific surface areas of KC and PIK were 16.81 m^2^ g^−1^ and 22.49 m^2^ g^−1^, respectively. The specific surface area of KC significantly increased after PI modification. The OH^−^ in the PI precursor will corrode and passivate the surface and interlayer silicon of kaolin, resulting in an increase in its specific surface area. In addition, the spatial hindrance of kaolin disperses PI colloidal particles, resulting in a decrease in the surface pore volume of kaolin. The pore volume of KC decreased from 0.1729 cm^3^ g^−1^ to 0.1340 cm^3^ g^−1^, as shown in Table S1. The pore size distribution results in Fig. 3c and d indicate that KC is mainly composed of micro pores and intermediate pores, which is beneficial for the loading of PI colloids.

The thermogravimetric image of the sample shows that as the temperature increases from 20 °C to 800 °C, the weight loss of KC and PIK mainly concentrates in three stages (Fig. S4). The quality reduction in stage I (40–100 °C) is attributed to the loss of bound water in KC and PIK. In the stage II (100–380 °C), only PIK presented a significant weight loss, which was mainly attributed to the decomposition of the hydroxyl iron polymerization unit in PIK.^53^ The content rate of –OH/H_2_O in PIK could be calculated based on the thermal weight loss of all materials, as shown in Table S2. In the stage III, the structural water of kaolin began to be lost, and the structure of kaolin gradually shifts towards that of metakaolin. The proportion of structural water lost in this part was higher than in the stage I and II. The calculation results indicated that the –OH/H_2_O content of PI_0.26_K was higher than that of other PIK samples, indicating that polyhydroxy iron modification could increase the –OH content on the surface of kaolin, thereby improving the utilization rate of iron. However, the continuous increase of iron ions concentration did not increase the –OH content in PIK. Among all PIK samples, the weight loss of PI_0.26_K was the highest, which indicated that the state formed by the polymerization of Fe ions and –OH is the most stable, and could polymerize with kaolin to form relatively stable PIK sample. The modification of PI can stabilize the presence of iron and increase the hydroxyl content on the surface of kaolin. Subsequently, the research on adsorption performance also confirmed this point.

### 2.2. The influence of preparation conditions on the adsorption capacity of PIK adsorbent

**Fig. 4:**
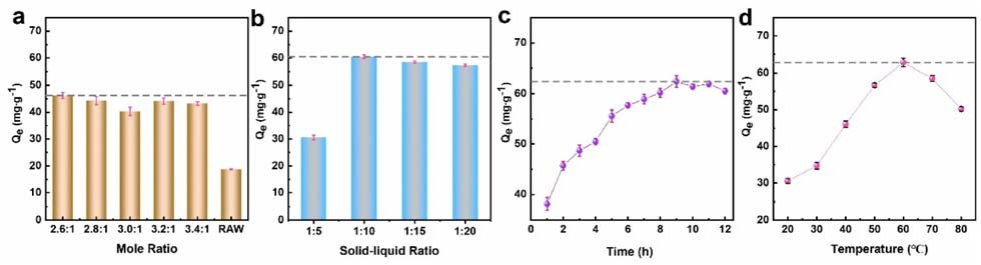
The influences of preparation conditions of PIK ((a) molar ratio of reagents, (b) solid–liquid ratio, (c) stirring time, (d) stirring temperature) on the adsorption of DB38 by PIK.

Fig. 4a shows the effect of PI prepared with K_2_CO_3_ and Fe (NO_3_)_3_ at different concentration ratios on the adsorption capacity of PIK. From Fig. 4a, it can be seen that when the concentration ratio of K_2_CO_3_ to Fe (NO_3_)_3_ is 1 : 2.6, the equilibrium adsorption capacity (*Q*_e_) of PIK for DB38 dye reaches a peak of 46.2 mg L^−1^ and then shows a decreasing trend. This was attributed to excessive hydrolysis of Fe^3+^ and the formation of hydroxide precipitates. The ratio of K_2_CO_3_ and Fe(NO_3_)_3_ concentrations directly influenced the stability of the PI precursor and the adsorption performance of PIK. Therefore, the optimal concentration ratio for preparing PIK was selected as 1 : 2.6.

Since PIK was stabilized through the hydrogen bonds and electrostatic interactions between the precursor PI and KC, the solid–liquid ratio of KC to PI determined the stability and adsorption capacity of PIK. The effect of the solid–liquid ratio of KC to PI on the adsorption performance of PIK was depicted in Fig. 4b. As could be seen in Fig. 4b, the *Q*_e_ for DB38 of PIK manifested an increase, ascending from 30.1 mg g^−1^ to 60.2 mg g^−1^, when the ratio of KC to PI increased from 1 : 5 to 1 : 10. This could be attributed to the thorough binding of PI to the surface sites of KC. Subsequently, increasing the solid–liquid ratio did not improve *Q*_e_. This is attributed to the aggregation and accumulation of PI precursors on the surface of kaolin, resulting in a decrease in *Q*_e_. Therefore, the solid–liquid ratio of KC to PI is selected as 1 : 10.

To ensure the complete combination between the precursor PI and KC without the formation of hydroxide precipitates, the influence of the requisite preparation time of the precursor PI on the adsorption performance of PIK was investigated in detail, as depicted in Fig. 4c. When the preparation time for the precursor PI reached 9 h, a stable PI was formed, and PIK prepared under such conditions exhibited the best adsorption performance for DB38, with a *Q*_e_ of 62.5 mg g^−1^. This demonstrated that the stability of the PI and the adsorption performance of PIK were correlated with the duration of ion hydrolytic polymerization. Consequently, a preparation time of 9 h for the precursor PI was selected.

It is well known that Fe^3+^ exists in different forms in water, and elevated temperature can induce a higher degree of Fe^3+^ hydrolysis, which makes it prone to form Fe(OH)_4_^−^ hydroxide precipitates and be unable bind stably with negatively charged KC.^54^ Thus, the temperature during the preparation of PI plays a crucial role within the hydrolytic polymerization process. To ensure the stable existence of the PI in the form of colloidal Fe(OH)_2_^+^ and to facilitate the thorough binding between PI and KC, the preparation temperature of PI was investigated. As presented in Fig. 4d, there were slight differences in the adsorption performance of PIK for DB38 at different temperatures. When the preparation temperature of the precursor PI reached 60 °C, PI had already stabilized, and the PIK prepared under such conditions exhibited the optimum adsorption performance for DB38, with a *Q*_e_ of 62.6 mg g^−1^. When the preparation temperature continued to rise, hydroxide precipitates were formed, leading to a decrease in the adsorption performance of PIK for DB38. Therefore, a preparation temperature of 60 °C for the precursor PI was chosen.

### 2.3 The influence of system environment on the adsorption performance of PIK adsorbent

**Fig. 5:**
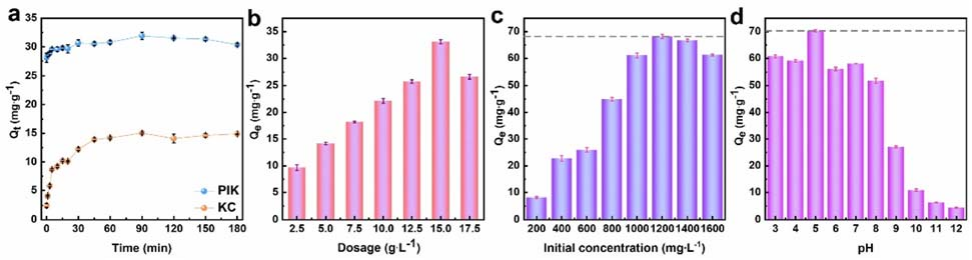
The influence factor ((a) adsorption time, (b) amount of adsorbent added, (c) initial concentration of dye solution, (d) pH of dye solution) on the adsorption of DB38 by PIK.

To ensure that PIK undergoes a complete reaction with DB38 to attain optimal adsorption performance, the optimum adsorption time is requisite. From the time-dependent profiles of KC and PIK shown in Fig. 5a, it could be observed that PIK exhibited a significantly superior adsorption capacity (*Q*_t_) for 500 mg L^−1^ DB38 comparison to KC. At 298 K, during the initial stages, *Q*_t_ for DB38 rapidly escalated with the augmentation of adsorption time, reaching 30.2 mg g^−1^ at 40 min, indicating a relatively high adsorption rate. When the time reached 90 min, *Q*_t_ became 33.6 mg g^−1^, and the adsorption rate decelerated, reaching equilibrium. Proceeding to increase to 180 min, *Q*_t_ slightly decreased, with overall negligible variation. Meanwhile, the adsorption capacity of KC for DB38 is 14.60 mg g^−1^. Therefore, the optimal adsorption time was 90 minutes.

Given the cost-effectiveness of adsorbents, it is necessary to determine the optimal dosage of adsorbents during application. The influence of PIK dosage on DB38 adsorption was shown in Fig. 5b. At an identical DB38 concentration of 500 mg L^−1^, the *Q*_e_ progressively increased with the augmentation of the PIK. The adsorption capacity of KC for DB38 is not as good as PIK, which may be due to the smaller specific surface area of KC. The modification of polyhydroxy iron augmented the specific surface area of KC (Table S1), thereby resulting in a significant elevation in the adsorption capacity of PIK for DB38 and providing new binding sites for DB38. PI played a crucial role in adsorbing DB38 due to its abundant hydroxyl functional groups. When the dose attained 15.0 g L^−1^, *Q*_e_ reached a maximum value of 32.8 mg g^−1^. Subsequently, at a dose of 17.5 g L^−1^, *Q*_e_ slightly declined, which could be attributed to the aggregation and incomplete utilization of PIK. Therefore, the optimal dose for PIK is 15.0 g L^−1^.

Fig. 5c showed the adsorption performance of PIK for DB38 with different initial concentrations. When the initial DB38 concentration increased from 200 mg L^−1^ to 1200 mg L^−1^, the *Q*_e_ of PIK for DB38 increased from 8.1 mg g^−1^ to 68.0 mg g^−1^. This occurred because the adsorption sites on the surface of PIK were perfectly congruent with the DB38 molecules in the solution, thereby elevating the value of the maximum adsorption capacity *Q*_e_ of PIK. When the concentration continued to increase to 1600 mg L^−1^, *Q*_e_ underwent a slight decrease (Fig. S5 and S6). This was attributed to the saturation of the adsorption sites on PIK surface and the significant aggregation of a considerable amount DB38 on the PIK surface, thereby leading to a decrease in *Q*_e_. Therefore, the optimal initial DB38 concentration is selected as 1200 mg L^−1^.

The pH value of the solution constitutes one of the significant factors influencing the adsorption performance of PIK. As shown in Fig. 5d, the adsorption capacity of PIK for DB38 increased and subsequently decreased with increment of the pH value. When the solution pH was 5.0, *Q*_e_ reached its maximum value of 70.26 mg g^−1^. With the augmentation of pH, *Q*_e_ gradually diminished. At pH 7.0, *Q*_e_ slightly increased. Continuing to increase the solution pH value, *Q*_e_ then decreased. This potentially attributed to, in the alkaline environment, the precipitation of Fe^3+^ or oxidation and degradation of the DB38 solution. The protonation of PIK intensified with increasing acidity, and the presence of Fe^3+^ on the surface of KC significantly affected the positive charge density on the KC surface. Therefore, the electrostatic force on the dye anions is enhanced, leading to an increase in *Q*_e_. The experiment results determined that the pH_pzc_ of PIK was 5.5. If the pH of the dye exceeded 6.0, the PIK surface would carry a negative charge, resulting in electrostatic repulsion with the dye anions, which is not conducive to the removal of the dye.^53^ Furthermore, it was discovered that *Q*_e_ reached its maximum at pH = 5.0, which indicated that in addition to electrostatic interactions, there were other interactions, such as hydrogen bond, existing. The abundant –OH and M–OH on the surface of PIK could form hydrogen bonds with functional groups such as –NH_2_, –OH, –SO_3_^−^, –N=N–, and benzene rings in the DB38 dye molecules. Under weakly acidic conditions, PIK achieved a higher *Q*_e_ for DB38.

Table S3 presented the pH alterations in the solution prior to and subsequent to adsorption. It was observed that when PIK adsorbed DB38 dye at different pH levels, there was a negligible variation in the pH before and after adsorption in the low pH range. This was attributed to the fact that the DB38 solution itself underwent protonation reactions with H^+^ ions and was associated with the stability of PIK. As the solution pH value increased, the competitive adsorption among the –NH_2_ groups in DB38, H^+^ ions, and the Fe^3+^ ions on the surface of PIK gradually decreased. Taking into account the overall adsorption performance, pH = 5.0 was selected as the optimal adsorption pH.

In conclusion, the optimal conditions for the adsorption of DB38 by PIK were as follows: the adsorbent dosage of 15.0 g L^−1^, the initial dye concentration of 1200 mg L^−1^, the solution pH of 5.0, and the adsorption time of 90 min. At this point, the adsorption capacity of PIK for DB38 is 5 times that of KC.

### 2.4. Mechanism of PIK adsorption of DB38

#### 2.4.1. Kinetic studies of adsorption

**Fig. 6:**
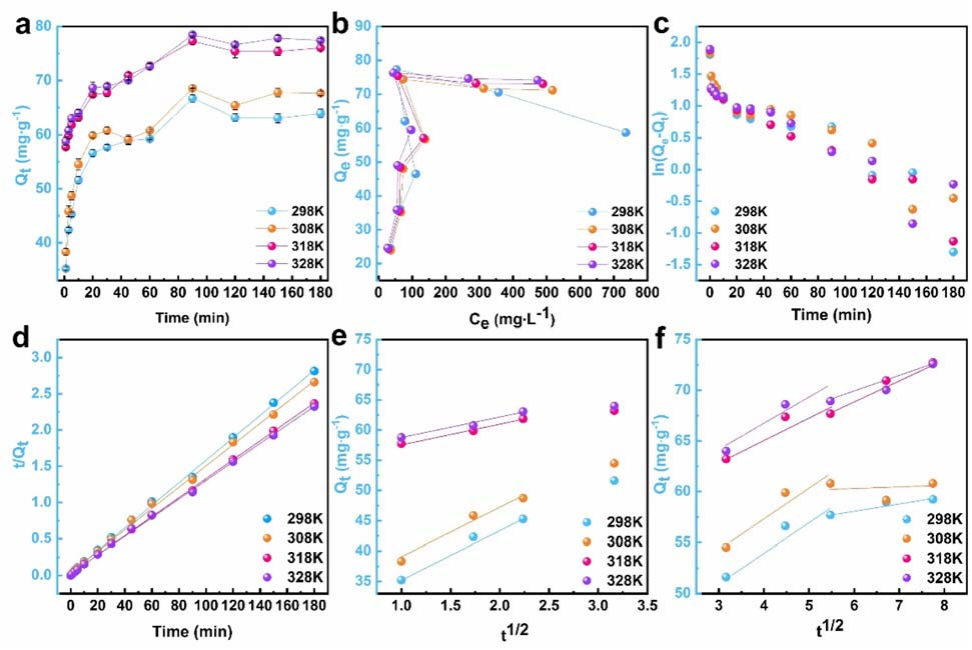
PIK adsorption kinetics curve (a), thermodynamic curve (b) and PFO (c), PSO (d) fitting curves, as well as intra particle diffusion fitting curves (1–10 min) (e) and (10–60 min) (f) for DB38.

For the purpose of in-depth exploration of the adsorption mechanism of PIK for DB38 dye molecules, pseudo first and pseudo second order models were adopted to fit the kinetic experimental data at different temperatures (298–328 K), the results of which were presented in Fig. 6.

As shown in Fig. 6a, at four different temperatures, namely 298 K, 308 K, 318 K and 328 K, respectively, PIK exhibited a comparable tendency in *Q*_e_ for DB38. At 298 K, *Q*_t_ reached 45.86 mg g^−1^ within the initial 3 min, indicating a rapid adsorption process. This was attributed to the fact that at the commencement of adsorption, the concentration of DB38 in the solution is elevated, leading to a rapid diffusion of DB38 molecules to the surface of PIK, thereby resulting in a higher adsorption rate. Subsequently, in the 3–20 min stage, in contrast to the 0–3 min stage, the rate gradually decelerated, and at 20 min, *Q*_t_ for DB38 reached 59.91 mg g^−1^. After 90 min, although a marginal decrease occurred, *Q*_t_ remained relatively stable, indicating that the adsorption sites on PIK gradually decrease over time, and the adsorption rate significantly decelerated. As shown in Fig. 6b, the change trend of *Q*_t_ was generally analogous across different temperatures ranging from 298 to 328 K. Furthermore, with the escalation of temperature, *Q*_t_ slightly increased, indicating that the adsorption process exhibited an endothermic characteristic, and the higher temperature expedited the movement of DB38 ions in the solution, enhancing their opportunities of contact with PIK and facilitating their adsorption more smoothly.^55^

The obtained pseudo-first-order (PFO) and pseudo-second-order (PSO) kinetic parameters, along with the corresponding correlation coefficients, were shown in Table 1. When combining Fig. 6c and d and Table 1, it could be noted that upon comparing the linear correlation coefficients *R*^2^ at different temperatures, the experimental data did not fully conform to the PFO adsorption model (*R*^2^ ≤ 0.9222), but were rather more appropriately described by the PSO adsorption model (*R*^2^ ≥ 0.9984). This suggested that the entire adsorption process involved chemical adsorption, and there existed chemical bonding and electron sharing between PIK and DB38. Moreover, *Q*_e_ increments with the rise in temperature, and the experimental equilibrium adsorption amount *Q*_exp_ was proximate to the theoretical equilibrium adsorption amount *Q*_ec_ calculated by the PSO kinetic model. The aforementioned results indicated that the PSO kinetic model offered a more accurate description of PIK adsorption process on DB38 at different temperatures, and this process exhibited endothermic characteristics.

**Table 1:** Kinetic parameters for the adsorption of dye DB38 onto PIK

*T*/K	Pseudo-first-order				Pseudo-second-order		
	*Q*_exp_/mg g^−1^	*k*_1_/min^−1^	*Q*_ec_/mg g^−1^	*R* ^2^	*k*_2_/g mg^−1^	*Q*_ec_/mg g^−1^	*R* ^2^
298	67.64	0.0228	11.94	0.9222	0.00629	68.03	0.9984
308	75.94	0.0260	24.42	0.8807	0.00569	75.19	0.9991
318	76.02	0.0288	21.21	0.8583	0.00787	76.33	0.9994
328	77.41	0.0260	21.83	0.6332	0.00640	78.13	0.9995

The intra-particle diffusion model was also employed to illustrate the diffusion mechanism within the adsorption process.^53,56^ The adsorption processes consist of three phases, namely the rapid adsorption phase, the internal diffusion phase, and adsorption equilibrium phase, respectively. To explore the intra-particle diffusion of PIK during the adsorption of DB38 and determine the controlling stage of adsorption rate, the plot of *Q*_t_ against *t*^1/2^ was employed. Linear curves traversing through the origin while maintaining an outstanding linear relationship were observed. This facilitated the determination of whether the adsorption process was governed by intra-particle diffusion.^53,57^ It could be observed from Fig. 6e and f that the adsorption process encompassed three stages. At different temperatures, during the initial 0–5 min of adsorption, DB38 rapidly diffused to the surface of PIK under the influence of concentration gradients, representing the rapid external diffusion stage. In the fast adsorption phase, the *K*_1_ value reached its peak, indicating that the adsorption capacity was most potent when DB38 rapidly diffused onto the outer surface of PIK.^53,58^ Within 5–10 min timeframe, due to the presence of chemical adsorption, DB38 gradually permeated into the micropores and adsorption sites within PIK, thereby resulting in an increasing *Q*_e_. In the internal diffusion phase, intra-particle diffusion emerged as the predominant factor constraining the adsorption rate. During the 10–60 min interval, DB38 molecules progressively diffused into the adsorption sites within PIK, attaining equilibrium gradually. The entire process was governed by intra-particle diffusion in the latter two stages.

#### 2.4.2. Thermodynamic investigation, separation factor evaluation and thermodynamic analysis

**Fig. 7:**
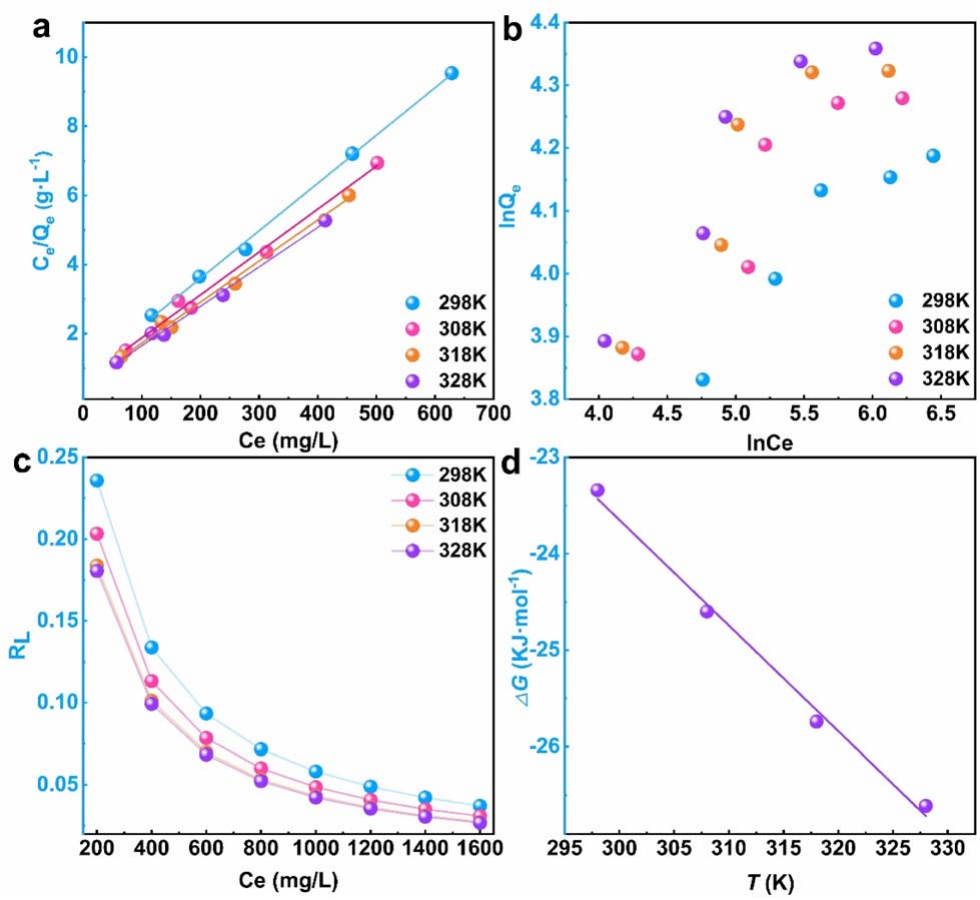
Langmuir (a) and Freundlich (b) isotherm adsorption fitting curves, equilibrium parameters and concentration relationship (c) and Gibbs free energy (d) of PIK adsorption on DB38.

The Langmuir and Freundlich isotherm adsorption models were utilized to investigate the adsorption behavior of PIK towards DB38 dye. The isothermal sorption curves and fitting parameters of PIK for DB38 were respectively presented in Fig. 7 and Table 2. The fitting results and data of the adsorption isotherm of PIK adsorbing DB38 at different temperatures were shown in Fig. 7a, b and Table 2. As the initial concentration of DB38 (*C*_0_) increased, *Q*_e_ increased accordingly, and also exhibited a minor increment with temperature.

**Table 2:** Results obtained from fitting Langmuir and Freundlich models for DB38 sorption by PIK

*T*/K	Langmuir isotherm			Freundlich isotherm		
	*Q*_max_/(mg g^−1^)	*b*/(L mg^−1^)	*R* ^2^	*n*	*K*_F_	*R* ^2^
298	72.62	0.0162	0.9974	4.78	17.78	0.8547
308	80.45	0.0195	0.9885	4.41	18.60	0.7950
318	84.03	0.0222	0.9893	4.17	18.65	0.7725
328	87.41	0.0227	0.9925	4.04	18.75	0.8301

From the data results presented in Table 2, it could be observed that the adsorption behavior of PIK for DB38 was more aptly characterized by the Langmuir model (*R*^2^ ≥ 0.9885), which markedly surpassed the Freundlich isotherm model in fitting the experimental data (*R*^2^ ≥ 0.8547). At different temperatures, the maximum adsorption capacity (*Q*) obtained experimentally was highly proximate to the saturation adsorption capacity (*Q*_max_), which indicated that the adsorption of DB38 on PIK was monolayer adsorption.^53,59,60^ The increment of Langmuir adsorption coefficient (*b*) with temperature indicated an endothermic adsorption process, which was in accordance with the kinetic results. The highest *b* value of PIK indicated its potent adsorption effect and high adsorption capacity. Meanwhile, upon reanalyzing the Freundlich isotherm adsorption model, it was found the progress of the adsorption process significantly depends on the *n* value. Specifically, when *n* ≥ 2, the adsorption proceeds was carried out easily; conversely, *n* < 2 made this process difficult to proceed. Within the experimental parameters, where temperatures ranged from 298 K to 328 K, all observed *n* > 2, signifying the ease of the adsorption reaction under these conditions. Compared with the adsorption capacity of other materials (Table S4 and S5), it suggested that PIK was a promising environmental material for the remediation of DB38.

Based on the conclusions derived from the separation factor *R*_L_ of the Langmuir model, the adsorption process tended to proceed smoothly when 0 < *R*_L_ < 1. It could be seen from Fig. 7c that the equilibrium coefficients *R*_L_ of PIK for DB38 at different temperatures were within the range of 0 to 0.024, which indicated that the adsorption process of DB38 onto PIK was easily achievable, signifying outstanding adsorption performance of PIK.

Further investigation, as shown in Fig. S7 and Table S6, revealed that the Temkin isotherm constant *A*_T_ gradually decreased with increasing temperature. As the surface coverage of DB38 increased, the adsorption binding affinity between the PIK adsorbent and DB38 weakened, and no significant improvement in adsorption capacity was observed. A higher *A*_T_ value indicates stronger initial binding between the adsorbent and the contaminant, typically associated with more prominent chemical interaction characteristics. In summary, the relatively high *A*_T_ values obtained from the fitting in this work suggest that the prepared PIK adsorbent exhibits a strong initial binding affinity for DB38 dye molecules, providing solid theoretical parameter support for its excellent adsorption performance. Additionally, when significant repulsive interactions exist between adsorbed molecules, the adsorption of subsequent molecules becomes more difficult, which is also reflected by a larger *b*_T_ value. The heat of adsorption (Δ*H*) decreases linearly with an increase DB38 coverage on the PIK surface. This is because the highest-energy, most reactive sites are occupied first, and as these sites become saturated, subsequent molecules can only adsorb onto lower-energy sites with weaker attraction.

The adsorption experimental data of PIK for DB38 was fitted employing the Langmuir isotherm model, which was applied to calculate the Δ*G*.^61,62^ The relationship between Δ*G* and *T* was depicted in Fig. 7d, and the calculated results of Δ*G* were shown in Table 3. Comprehensively considering these data, it could be concluded that at different temperatures (298–328 K), the PIK adsorption free energy Δ*G* for DB38 was consistently negative, ranging from –26.61 to –23.34 kJ mol^−1^, which indicated that the adsorption of PIK for DB38 was a spontaneous process.^63,64^

**Table 3:** Thermodynamic parameters of PIK adsorption on DB38

*T*/K	298	308	318	328
Δ*G* (kJ mol^−1^)	–23.34	–24.60	–25.74	–26.61
Δ*H* (kJ mol^−1^)	9.36			
Δ*S* (J mol^−1^ K^−1^)	110.00			

The positive Δ*H* of PIK was greater than 0, being 9.36, indicated that the adsorption process was an endothermic reaction (involving an increase in internal energy). Therefore, an increase in temperature was conducive to promoting the adsorption of PIK for DB38. The positive Δ*S* values for PIK suggested that the adsorption process increased the disorder at the solid–liquid interface.^65^

#### 2.4.3. The dye possible adsorption mechanism of PIK

**Fig. 8:**
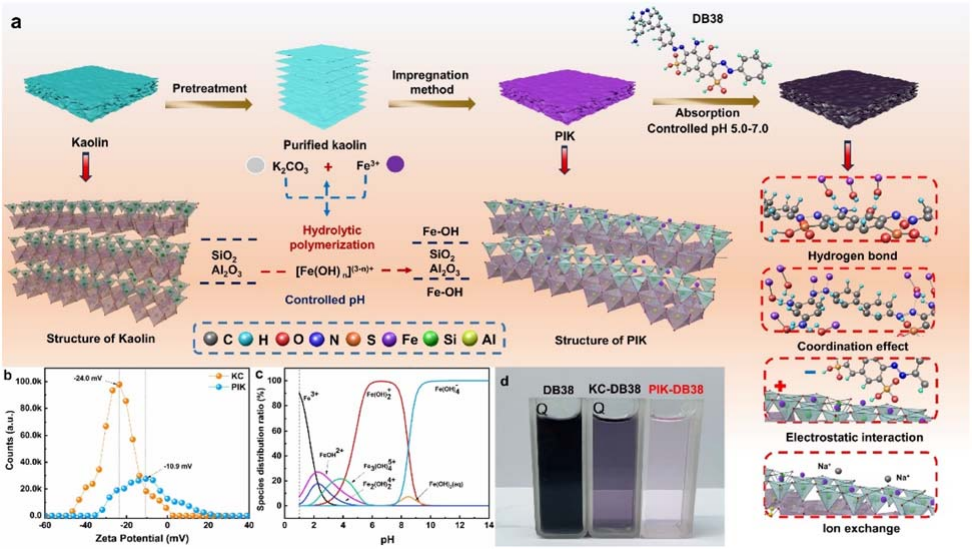
The adsorption mechanism of PIK for DB38 dyes (a), zeta potential (b), distribution of iron ion forms (c) and comparison of DB38 adsorption effects between KC and PIK (d).

In order to reveal the adsorption mechanism of DB38 on PIK (Fig. 8a), the physicochemical properties of PIK before and after adsorption of DB38 are comparatively analyzed through SEM, BET, EDS, Zeta potential, FTIR, and XPS. SEM images manifested that the surface morphology and pore structure of KC and PIK remained unaltered subsequent to the adsorption of DB38 (Fig. S8a, b, S9a, b and S10), which indicated that the KC and PIK owned high stability and persistence.

The BET and BJH results indicate a decrease in the specific surface area of both KC and PIK, suggesting that a large amount of DB38 is attached to the pores of KC and PIK (Table S1 and Fig. S11a–d). At the same time, the average pore size and pore volume of KC also decreased, while the average pore size and pore volume of PIK increased, indicating the existence of partial ion exchange processes. Combining the results of fitting the adsorption kinetics curves with the internal diffusion model revealed that PIK exhibited a very strong adsorption capacity in the first stage and continued to have a strong adsorption capacity in the second stage of the adsorption process. These findings collectively demonstrate that pore filling played a crucial role in the adsorption process.^53^ In addition, ion exchange also plays a role in adsorption, where Fe^3+^ ions originally bound to the surface of KC are partially exchanged by DB38 molecules.

According to the Fig. 8b, it can be seen that in a neutral environment, the KC zeta potential was –24.0 mV and the surface charge was in a highly negative state, which signified the existence of a large amount of exposed negative charges in the layered structure of the KC surface. After modification with polyhydroxy iron, the zeta potential of the PIK sample altered to –10.9 mV and the surface negative charge was diminished, which indicated that the modification of polyhydroxy iron changed the surface charge environment. When pH ≥ 5.5, the surface charge environment of PIK shifted from a positive charge attribute to a negative charge attribute, suggesting that a relative reduction of H^+^ ion in the environment would induce a change in the surface charge attribute of PIK. The morphology of 0.1 mol L^−1^ Fe^3+^ at diverse pH levels was simulated and calculated by employing Visual MINT EQ 3.1 software,^66^ as presented in Fig. 8c. It can be seen from the figure that Fe(OH)_2_^+^ was predominantly stable within the pH range of 5.0–7.5. When KC combined with Fe(OH)_2_^+^, its surface potential increased significantly, rising from –24.0 mV to –10.9 mV. This form of iron exerts an efficacious role in modifying kaolin, engendering changes of the surface charge environment. This change is conducive for PIK to adsorb specific dye molecules through electrostatic interactions. Zheng *et al.*^54^ also affirmed through DFT calculations that the chemical adsorption capacity of iron hydroxy compounds follows the conclusion of [Fe(OH)]^2+^ > [Fe(OH)_3_] > [Fe(OH)_5_]^2–^ > [Fe(OH)_2_]^+^ > [Fe(H_2_O)_6_]^3+^ > [Fe(OH)_4_]^−^ > [Fe(OH)_6_]^3–^. This perspective also substantiates the reliability of our prepared PIK materials. The solution state of the DB38 dye adsorbed by the adsorbent is shown in Fig. 8d.

**Fig. 9:**
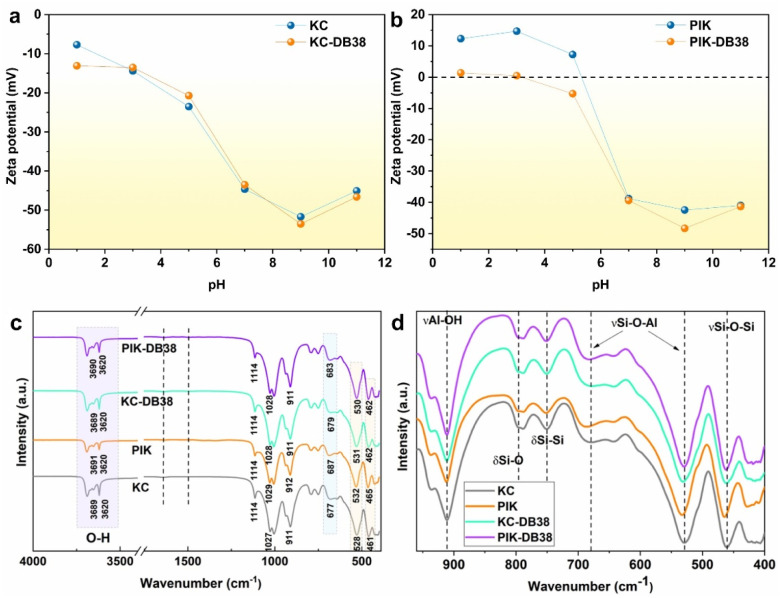
Zeta potential of KC and KC-DB38 (a), zeta potential of PIK after adsorption of DB38 (b). FT-IR (c) and (d) of KC, PIK, KC-DB38, and PIK-DB38.

The surface charge properties of KC and PIK at different pH stages were determined by zeta potential, and it was demonstrated that the surface charge of KC is negative, which is consistent with the conclusions provided in the literature.^67,68^ Moreover, the change in surface charge after KC adsorbs DB38 is not very significant (Fig. 9a), indicating that electrostatic adsorption is not the main factor in the adsorption process of KC adsorbing DB38. The solution pH affected the adsorption efficiency of DB38 by influencing the dissociation state of oxygenated groups on the surface of PIK (Fig. 5h). The PIK samples possessed a positive charge in a weakly acidic environment, while KC exhibited a negative charge property. After DB38 adsorption, the zeta potential of PIK became less positive (Fig. 9b), suggesting that DB38 adsorbed onto the PIK surface *via* electrostatic attraction, thereby reducing the positive surface charge of PIK.^69^

The FTIR results demonstrated that PIK exhibited a red shift at the –OH stretching absorption peak at 3691 cm^−1^ upon the adsorption of DB38, which indicated that DB38 was adsorbed on the PIK surface by hydrogen bonding (Fig. 9c and d).^70^ Simultaneously, the Si–O–Al bent adsorption peaks at 532 cm^−1^ and 687 cm^−1^ displayed a red shift, indicating the contribution of the aluminum oxide layer to the adsorption of DB38. This might be due to the coordination effect between DB38 and Fe bound to the aluminum oxide layer.^71^ The Si–O–Si bending vibration absorption peak at 465 cm^−1^ has also shifted, which suggested that the silicon oxide layer of PIK also contributed to the adsorption of DB38 to a certain extent.^72^

**Fig. 10:**
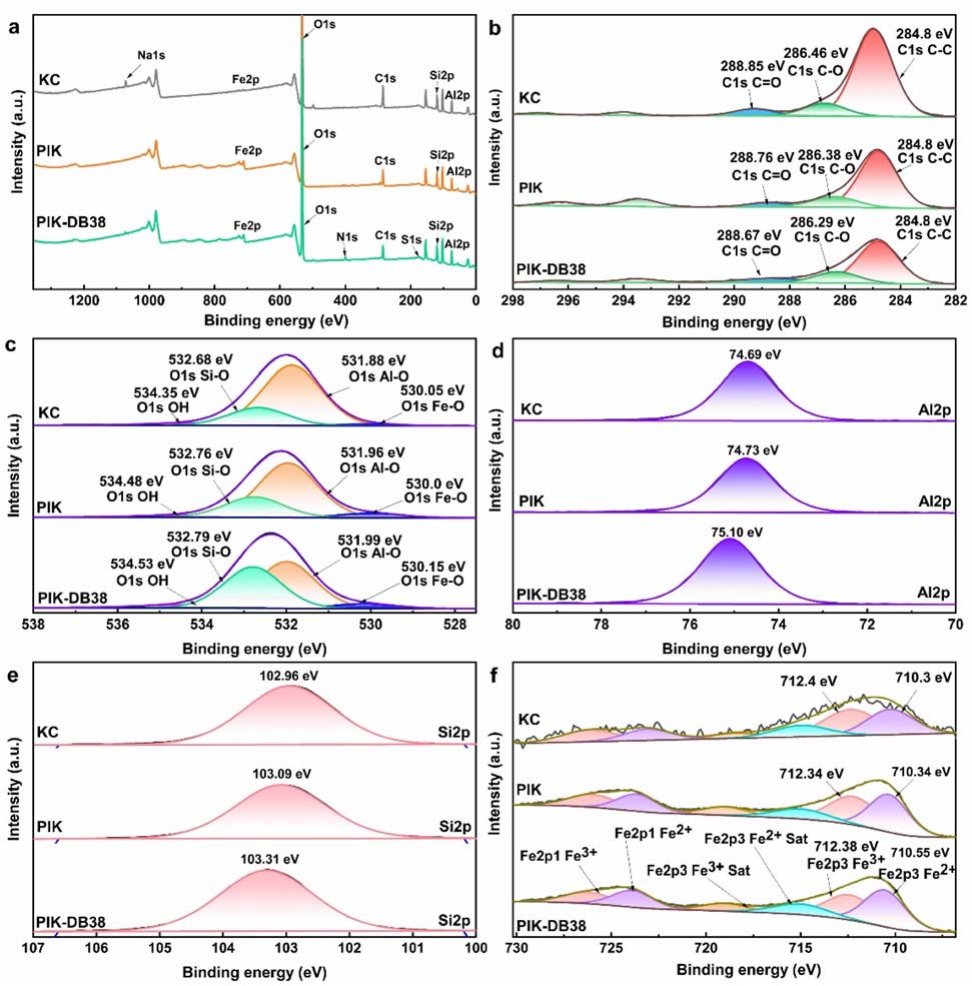
XPS full spectrum of (a) KC, PIK and PIK-DB38, high-resolution spectrum: C 1s (b), O 1s (c), Al (d), Si (e) and Fe (f).

In accordance with the results depicted in Fig. 10a, the characteristic peaks corresponding to C 1s, as well as those of O 1s, Al 2p, Si 1s, and Fe 2p, were observed in the XPS spectra of KC, PIK and PIK-DB38. After PIK adsorbed DB38 dye, the positions and intensities of the aforementioned characteristic peaks in the XPS spectrum underwent changes, and characteristic peaks of S 1s and N 1s also appeared in the sample. The XPS results showed a significant increase in the elemental contents of N and S on the surface of PIK after the adsorption of DB38 (See the Table S7 for details). This increase in elemental contents can be attributed to the high N and S contents in DB38, indicating that DB38 was adsorbed on the surface of PIK. After treatment with PI, the Na 1s peak of KC was disappeared, indicating the occurrence of ion exchange between Na^+^ and Fe^3+^ ions in the kaolin soil layer.

The high-resolution XPS spectrum of C 1s in Fig. 10b revealed a characteristic peak with a binding energy of 284.8 eV, which was attributed to the organic impurities in the KC itself or the C atoms in the residual free ethanol during the washing process of PIK.^73^ Upon the adsorption of DB38 by PIK, the content of C–C decreased from 9.72% to 6.87% (See the Table S7 for details). The binding energy of the C–C peak remained unaltered, while the binding energy of the C–O bond and C=O bond decreased respectively from 286.38 eV to 286.29 eV and 288.76 eV to 288.67 eV. This indicates that PIK obtained some electrons from CO_3_^2−^ during the adsorption of DB38.

It can be discerned from Fig. 10c that the O 1s in Al–OH of KC had a binding energy of 531.88 eV. After PI modification, the binding energy rose to 531.96 eV, indicating the formation of Al–O–Fe or that some Fe ions replaced Al ions. Correspondingly, the absorption peak of Al in Fig. 10d also shifts towards higher binding energy. Upon the adsorption of DB38 by PIK, the binding energy rose to 531.99 eV and the peak area decreased from 40.43% to 29.67%, which indicated that the –OH groups on the surface of PIK were replaced by dye anions during the adsorption process (See Table S7 for details).^74^ It can be observed from the high-resolution XPS spectrum of Al 2p in Fig. 10d that the peak with a binding energy of 74.73 eV was the Al–O peak in PIK.^75^ Upon the adsorption of DB38 by PIK, the binding energy shifted to 75.1 eV. This indicated that adsorption also occurred on the Al–O layer on the PIK surface, resulting in an increase in binding energy. These changes implied electron transfer and coordination between Fe and DB38 on the surface of PIK. At the same time, a reaction occurred between Si and Al on the surface of kaolin and DB38 molecules. There were hydrogen bonding and ion exchange interactions between functional groups such as –OH and Fe–OH on the surface of PIK and functional groups such as –N=N–, –NH_2_, –OH, and –SO_3_^2−^ of the DB38 dye molecule.

The high-resolution XPS spectrum of Si 2p in Fig. 10e disclosed that the binding energy of Si in KC is at 102.96 eV, almost in accordance with the standard binding energy position (103.0 eV). After modification with PI, it underwent a shift towards higher binding energy, indicating that PI also had an impact on the surface of silicon oxide crystals. When the PIK adsorbed the DB38, the Si binding energy shifted to 103.31 eV, which indicated that electrons were lost due to chemical interactions. In combination with the fact that the binding energy of Si–O in Fig. 10c increased from 532.76 eV to 532.79 eV, suggesting that the Si–O layer also played a certain role in the adsorption process.

Fig. 10f presented the high-resolution XPS spectrum of Fe 2p. It can be seen that the Fe 2p_3/2_ spectrum in PIK split into two peaks at binding energies of 712.4 eV and 710.3 eV, while the Fe 2p_1/2_ spectrum also bifurcated into two peaks at 723.2 eV and 727.2 eV. These results indicated the concurrent existence of Fe^3+^and Fe^2+^ in PIK.^76–78^ Specifically, the peaks at 710.0 eV and 723.2 eV were attributed to Fe^2+^, while the peaks at 712.5 eV and 727.2 eV were attributed to Fe^3+^.^79,80^ After adsorption of DB38 dye by PIK, the binding energy and peak area of iron element underwent alterations. The binding energies of Fe^2+^and Fe^3+^ increased by 0.21 eV and 0.17 eV, respectively, and the peak areas both decreased (See Table S7 for details). This proved that electron transfer and coordination occurred in Fe element after adsorption of DB38 by PIK. Meanwhile, the variation in the binding energy of Fe–O from 530.0 eV to 530.15 eV before and after adsorption of DB38 by PIK in Fig. 10c also corroborated the role of Fe–OH in adsorption. The presence of Fe^2+^ may be due to the use of ethanol in the preparation of PI modified kaolin.

In summary, we hold that the principal adsorption mechanisms of DB38 by PIK involved pore filling, ion exchange, electrostatic attraction, coordination effect, and hydrogen bond.

### 2.5. Reusability

**Fig. 11:**
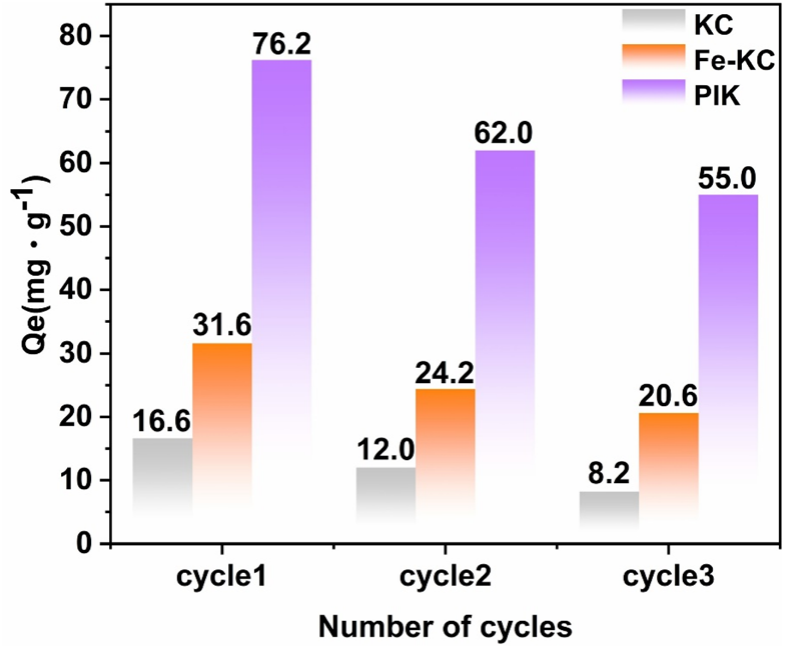
Adsorbents adsorption performance over multiple cycles of adsorption and regeneration.

The regeneration ability of the adsorbent is one of the crucial factors determining whether it can be widely applied in the industry.^81^ It could be seen from Fig. 11 that after three cycles of adsorption–desorption, the adsorption capacity of PIK decreased by 28%, whereas that of KC decreased by 50% after the first cycle. This also indicated that PIK was more resistant to adsorption and desorption, with chemical adsorption predominating. The adsorption capacity of Fe-KC prepared by impregnation the same concentration of Fe^3+^ was 22.2 mg g^−1^ after three cycles, which was only 41.5% of that of PIK. PIK retained 82% of its adsorption capacity after three cycles, thereby demonstrating its excellent reusability. According to the data in Table S8, the iron leaching amount increased significantly over three consecutive cycles. This phenomenon may be attributed to the repeated washing steps during adsorbent recovery and the alkaline environment (pH = 7.0–7.5) induced by the DB38 solution. The stability of PI compounds is highly reliant on the pH control during synthesis. PIK adsorbents prepared under weakly acidic conditions exhibited stronger adsorption performance. During the adsorption process, the Fe species on the adsorbent surface and within the pore structures were gradually lost due to continuous neutralization in the high-concentration DB38 medium, accompanied by charge neutralization with counter ions, ultimately resulting in a decline in adsorption performance. However, compared with Fe-KC, the PIK adsorbent exhibited a significantly lower iron leaching amount. This finding further corroborates that the adsorption capacity of PIK for DB38 is closely related to the stable retention of iron species.

## 3. Conclusions

A novel adsorbent with outstanding adsorption capacity, polyhydroxy iron-modified kaolin (PIK), is fabricated through a mild, green low-cost and mass-producible low-temperature liquid-phase approach. In comparison with the original kaolin, the adsorption capacity of obtained PIK for DB38 is significantly improved, reaching 76.6 mg g^−1^. Polyhydroxylated iron can modify the surface charge environment of kaolin, increase its pore volume, specific surface area, iron loading and content of –OH functional groups. Therefore, the adsorption capacity of PIK for DB38 is significantly elevated through pore filling, electrostatic attraction, hydrogen bonding, coordination effect and ion exchange. Furthermore, PIK maintains a relatively strong adsorption capacity and reusability under various environmental conditions (temperature and pH). Therefore, it is feasible to employ polyhydroxylated iron modified kaolin for the adsorption of waste dye pollutants, and this process holds considerable industrial application prospects.

Owing to its negative charge property, PIK exhibits different adsorption efficiencies for different types of dyes in solutions and especially shows outstanding adsorption performance for anionic azo dyes (Fig. S12 and S13, the structures of the compounds are listed in Table S9). This material not only exhibits excellent performance in the removal of organic pollutants, but also yields significant effects in the elimination of fluoride ions and phosphate ions. Therefore, the outcomes of this study also suggest that the composite modification of kaolin and its loading with other substances could potentially be the direction for the future development of green economy of kaolin material.

## Conflicts of interest

The authors declare no competing financial interest.

## Supplementary Material

RA-015-D5RA05883J-s001

## Data Availability

The authors confirm that the data supporting this article have been included as part of the manuscript. Supplementary information (SI) is available. See DOI: https://doi.org/10.1039/d5ra05883j.
