# Supplementary material for: Scalable synthesis of polyhydroxy iron-modified kaolin via low-temperature hydrolysis-polymerization for efficient adsorption of direct black 38
Source: RSC Adv. 2025 Nov 24;15(53):45678–89. doi: 10.1039/d5ra05883j (PMC12641248; doi:10.1039/d5ra05883j)
Supplement: RA-015-D5RA05883J-s001 [file RA-015-D5RA05883J-s001.pdf]

Supplementary Information (SI) for RSC Advances.
This journal is © The Royal Society of Chemistry 2025

Supplementary Information

Scalable Synthesis of Polyhydroxy Iron-Modified Kaolin via Low-Temperature Hydrolysis-Polymerization for Efficient Adsorption of Direct Black 38

Xi Duo[a, b, 1], Qingyi Qian[a, 1], Yunsong Zhang[a], Liangliang Bao[a, b], Qier Mu[c], Hui Yan[a, d, *] Latai Ga[e, *], Zhimei Zhong[a,b, *]

[a] College of Science, Inner Mongolia Agricultural University, Hohhot 010018, Inner Mongolia, China

[b] Inner Mongolia Key Laboratory of Soil Quality and Nutrient Resource, Hohhot 010018, Inner Mongolia, China

[c] College of Animal Science, Inner Mongolia Agricultural University, Hohhot 010018, Inner Mongolia, China

[d] College of Materials Science and Art Design, Inner Mongolia Agricultural University, Hohhot 010018, China

[e] School of Renewable Energy, Inner Mongolia University of Technology, Ordos 017010, China

[*] Corresponding authors. E-mail addresses:

yanhui@imau.edu.cn (Hui Yan),

galtai@imut.edu.cn (Latai Ga),

zhimeihappy@126.com (Zhimei Zhong).

[1,] These authors contributed equally to this work

Total Number of pages: 23

Total Number of tables: 9

1. Materials and methods

1.1 Materials and reagent

The main reagents used are $Fe(NO_3)_3·9H_2O$ (AR, Sinopharm chemical Reagent co.,Ltd.); K_2CO_3 (AR, Sinopharm chemical Reagent co.,Ltd.); Anhydrous ethanol (AR, Tianjin Zhiyuan Chemical Reagent Co., Ltd.); Kaolin (Hunan Shanlin Mineral Products Co., Ltd, 99.5%); DB38 (CP, λ_{Max}=590 nm, Mr=781.7, Tianjin Zhiyuan Chemical Reagent Co., Ltd.). The solutions are prepared using ultrapure water (18.25 MΩ·cm) as the solvent.

1.2 Preparation of polyhydroxy iron (PI)

According to our previous research results[1], the appropriate molar ratio of carbonate to $Fe(NO_3)_3$ concentration and appropriate stirring temperature can improve the degree of association and stability during the formation of polyhydroxy iron precursors. Based on the above factors, the concentration molar ratio of K_2CO_3 solution (0.1 $mol·L^{-1}$) to Fe $(NO_3)_3$ solution (0.26 $mol·L^{-1}$) is selected, and the stirring temperature of 60 °C is chosen as the preparation process temperature for polyhydroxy iron. The 100 mL K_2CO_3 solution is slowly added through a separating funnel into a 100 mL Fe $(NO_3)_3$ solution, and the resultant mixed solution is stirred for 9 h at 60 °C. Then, the solution is sonicated for 30 minutes, and finally aged at room temperature for 24 h to obtain the precursor of polyhydroxy iron, which is recorded as **PI**.

1.3 Preparation of polyhydroxy iron modified kaolin (PIK)

According to different solid-liquid ratios, a certain amount of kaolin is slowly added to the PI and continuously stirred at 60 °C for 9 h. After the reaction is completed, the obtained solution is sonicated for 30 minutes and then aged for 24 h at room temperature. The insoluble substance is separated from the suspension by centrifugation, and then washed multiple times with deionized water and anhydrous ethanol until the washing supernatant is nearly colorless. Subsequently, the product is

placed in a drying oven and dried at 60 °C for 8 h. Finally, the obtained product is passed through a 300-mesh sieve and ground, which is designated as polyhydroxy iron modified kaolin, denoted as **PIK**. The aqueous solutions of 0.10 mol/L K_2CO_3 mixed with varying concentrations of $Fe(NO_3)_3$ were designated as $PI_{0.05}$ to $PI_{0.50}$. The resulting PIK composites, prepared by combining these PI solutions with kaolinite, were correspondingly labeled as $PI_{0.05}K$ to $PI_{0.50}K$.

2. Characterization

The specific surface area and pore characteristics of kaolin (KC) and polyhydroxy iron modified kaolin (PIK) were analyzed at 77.3 K using the Brunuer-Emmett-Teller (BET) and the Barret-Joyner-Halenda (BJH) N_2 adsorption-desorption method (BEISHIDE 3H-2000PS1, China). The weight change (TGA) of kaolin clay (KC) and polyhydroxy iron modified kaolin (PIK) was measured by a thermogravimetric analyzer over the temperature range of 20–800°C at a heating rate of 20 °C min^{-1} under a N_2 purge (HENVEN HCT-4, China). The surface functional groups of kaolin clay (KC) and polyhydroxy iron modified kaolin (PIK) were measured using a Fourier Transform infrared (FTIR) spectrometer (PerkinElmer Spectrum Two, America). Scanning electron microscopy (SEM) coupled with energy-dispersive X-ray spectroscopy (EDS) was used to determine the surface morphology and elemental composition of kaolin clay (KC) and Polymer hydroxylated iron modified kaolin (PIK) (Hitachi Regulus 8100, Japan). Zetasizer was used to analyze the surface electrical properties of kaolin clay (KC) and polyhydroxy iron modified kaolin (PIK) under different pH conditions (Malvern Zetasizer Nano ZS-90, Britain). X-ray diffraction (XRD) analysis was performed to determine the surface crystal structure of kaolin clay (KC) and Polymer hydroxylated iron modified kaolin (PIK) (Tongda TD-3500, China), and the chemical composition and valence states of kaolin clay (KC) and polyhydroxy iron modified kaolin (PIK) were investigated by X-ray photoelectron spectroscopy (XPS, Thermo Scientific K-Alpha, America). ICP-OES (Agilent, 5800, USA) was used to detect Fe in the DB38 degradation solutions.

2.1 Material characterization

The surface morphology and chemical composition of kaolin were analyzed by means of spectroscopic techniques. The loading mass of hydroxyl groups on PIK was determined through thermogravimetric analysis.

2.2 Batch adsorption experiment

Varying amounts (0.05 to 0.35 g) of PIK are incorporated into 20 mL of 500 mg·L^{-1} DB38 solution (2.5 to 17.5 g·L^{-1}). The mixture is subjected to shaking at 120 rpm for 180 min at 25 °C to investigate the influence of kaolin dosage on the adsorption of DB38[2, 3].

Isotherms experiments are carried out by using 20 mL DB38 solution with a concentration ranging from 200 to 1600 mg·L^{-1} and 0.30 g of PIK. The mixtures are shaken at 180 rpm for 180 min. Kinetic adsorption experiments are performed by using 20 mL of 100 mg·L^{-1} DB38 solution and 0.30 g PIK. Sampling is conducted at different time intervals (from 1 to 180 min). The mixed solutions are filtered through a needle filter (water system, 0.45 μm) for further testing[4].

To explore the impacts of PIK on the adsorption performance of DB38 under various environmental conditions, 0.30 g PIK is added to 20 mL of 100 mg·L^{-1} DB38 solution. Subsequently, the adsorption efficiencies of DB38 by PIK are examined under multiple circumstances, namely the pH and temperature of background solution. The pH of the background solution is adjusted from 3 to 12 through the application of 0.1~1 mol·L^{-1} of HCl and NaOH[4]. Additionally, different temperature conditions are set at 298, 308, 318 and 328 K, respectively.

All adsorption experiments are performed in triplicate, and the samples of DB38 are analyzed by a UV-vis spectrophotometer (TU-1901, Beijing) at a wavelength of 590 nm. The adsorption quantity and removal rate of DB38 are calculated as follows [5, 6]:

$$R(\%) = \frac{c_0 - c_e}{c_0} \times 100\% \quad (1)$$

$$Qe = \frac{(c_0 - c_e)V}{m} \quad (2)$$

In the formula, R represents the dye removal rate, expressed as "%"; C_0 and C_e denote the initial and equilibrium concentrations of the dye prior to and after adsorption, respectively, measured in $mg \cdot L^{-1}$; Q_e designates the equilibrium adsorption capacity of the adsorbent, in $mg \cdot g^{-1}$; m indicates the dosage of adsorbent, in g; and V signifies the volume of the dye solution, in L.

2.3 Adsorption model

The adsorption kinetics of DB38 onto PIK are scrupulously modeled using the pseudo-first-order, pseudo-second-order and intraparticle diffusion frameworks, respectively. Meanwhile, the Langmuir and Freundlich isotherms are employed to fit the adsorption equilibrium data. A comprehensive thermodynamic investigation is carried out to illuminate the thermodynamic properties governing the DB38 adsorption process on PIK.

2.4 Regeneration and potential of PIK

To assess the reusability of the PIK material, three consecutive adsorption/desorption cycles are conducted. Initially, 0.30 g PIK is immersed in 20 mL of a 1200 $mg \cdot L^{-1}$ DB38 solution and subjected to agitated for 180 min to achieve adsorption. Subsequently, the DB38-laden sample is subjected to desorption via sonication in 50 mL of 0.01 $mol \cdot L^{-1}$ HCl solution maintained at 298 K for 30 min, and this step repeated until the eluent become colorless, in accordance with established protocols [7]. The desorbed PIK is then comprehensively dried and reused in subsequent rounds of adsorption experiments. This entire cycle, including adsorption, desorption and drying, is repeated a total of three times.

3. Adsorption models

3.1 Isotherms models

Langmuir and Freundlich models were applied to determine the adsorption characteristics of PIK. The two isotherm equations are provided as follows:

Langmuir model：

$$\frac{C_e}{Q_e} = \frac{1}{b \cdot Q_{\max}} + \frac{C_e}{Q_{\max}} \qquad \text{(S1)}$$

Freundlich model：

$$\ln Q_e = \ln K_F + \frac{1}{n} \ln C_e \qquad \text{(S2)}$$

In the equation, Q_e and Q_{max} are the actual adsorption capacity and theoretical saturated adsorption capacity at adsorption equilibrium, mg·g^{-1}; C_e is the adsorption equilibrium concentration, mg·L^{-1}; b is the Langmuir adsorption equilibrium constant, L·mg^{-1}; K_F is the adsorption equilibrium constant of Freundlich, L·mg^{-1}; n is the adsorption strength parameter.

$$Q_e = \frac{RT}{b_T} ln(A_T C_e) \qquad \text{(S3)}$$

A_T is the equilibrium binding constant related to the adsorption energy, L·g^{-1}; b_T is the Temkin constant related to the heat of adsorption, J·mol^{-1}; R is the ideal gas constant, 8.314 J/(mol·K^{-1}); and T is the absolute temperature, K.

3.2 Sorption kinetics

In order to investigate the adsorption mechanism of PIK on DB38, quasi-first-order and quasi-second-order kinetic models were used to fit the kinetic experimental data at different temperatures (298~328 K). Additionally, the Weber-Morris intraparticle diffusion model was employed to calculate and discuss the adsorption stages. The equation of the three kinetic models is represented as follows:

Pseudo-first-order model:

$$\log(Q_e - Q_t) = \log Q_e - \frac{k_1 - t}{2.303} \qquad \text{(S4)}$$

Pseudo-second-order model:

$$\frac{t}{Q_t}=\frac{1}{k_2\cdot Q_e^2}+\frac{t}{Q_e} \quad \text{(S5)}$$

Weber Morris Intra-particle diffusion model:

$$Q_t=k_i t^{1/2}+C \quad \text{(S6)}$$

In the formula, k_1 is the first order adsorption rate constant, L·min^{-1}; k_2 is the second order adsorption rate constant, g·(mg·min)$^{-1}$; Q_e is the equilibrium adsorption capacity, mg·g^{-1}; Q_t is the adsorption amount at the time when the removal rate is t, mg·g^{-1}; T is the adsorption time, min; C is the diffusion constant within the particle.

3.3 Thermodynamics model

Gibbs-Helmholtz equation was used to understand the thermodynamics of DB38 adsorption onto the PIK. Its equation can be expressed as below:

$$\Delta G =- RT\cdot lnK_L \quad \text{(S7)}$$

$$K_L = Q_e/C_e \quad \text{(S8)}$$

$$lnK_{L}=\frac{\Delta S}{R}-\frac{\Delta H}{RT} \quad \text{(S9)}$$

Where ΔH, ΔS, and ΔG are enthalpy, entropy, and Gibbs free energy, respectively; K_L is the Langmuir equilibrium constant, R is the gas constant (8.314 J (mol·K^{-1})) and T is the operating temperature in Kelvin.

3.4 Adsorption experiment on other dyes

To investigate the adsorption capacity of KC and PIK to other dyes in solution, 0.20 g of KC or PIK was added to 20 mL of 200 mg·L^{-1} dye (CR, DB38, MB, MG, RhB, and MO) solution, respectively. The pH of the background solution was adjusted with 1 mol·L^{-1} of HCl and NaOH. The mixture was shaken under 120 rpm for 180 min at 25 °C, after that the mixed solutions were filtered through a needle filter (organic system, 0.45 μm). The samples of CR, DB38, MB, MG, RhB, and MO were analyzed by a UV-Vis spectrophotometer (TU-1901, Shanghai) at a wavelength of 497, 590, 664, 618, 554, and 464 nm, respectively. All the adsorption experiments were carried out in triplicate.

Table S1. Specific surface area, pore volume, and mean pore diameter of PIK and KC before and after DB38 adsorption.

Materials	Specific Surface Area ($m^2\ g^{-1}$)	Pore volume ($cm^3\ g^{-1}$)	Average pore width (nm)	Most Frequent Pore Diameter (nm)
KC	16.81	0.1729	31.38	21.32
PIK	22.49	0.1340	19.63	19.64
KC-DB38	14.42	0.1532	30.07	20.59
PIK-DB38	21.71	0.1635	25.21	19.28

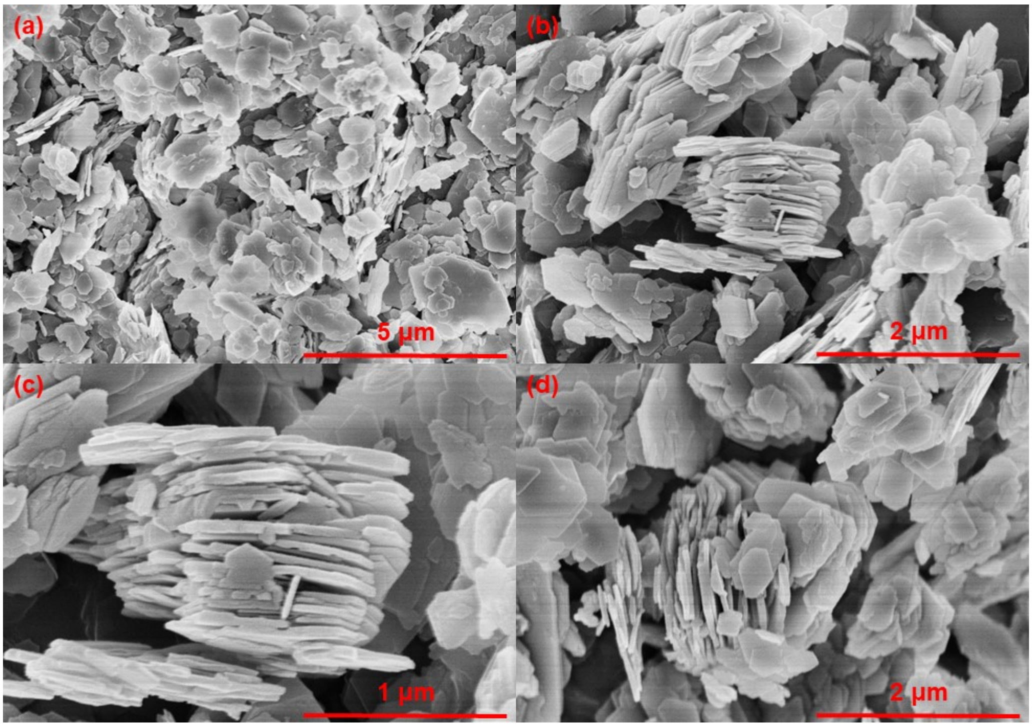

Fig. S1: Scanning electron microscopy images of kaolin Clay (KC) at different magnification

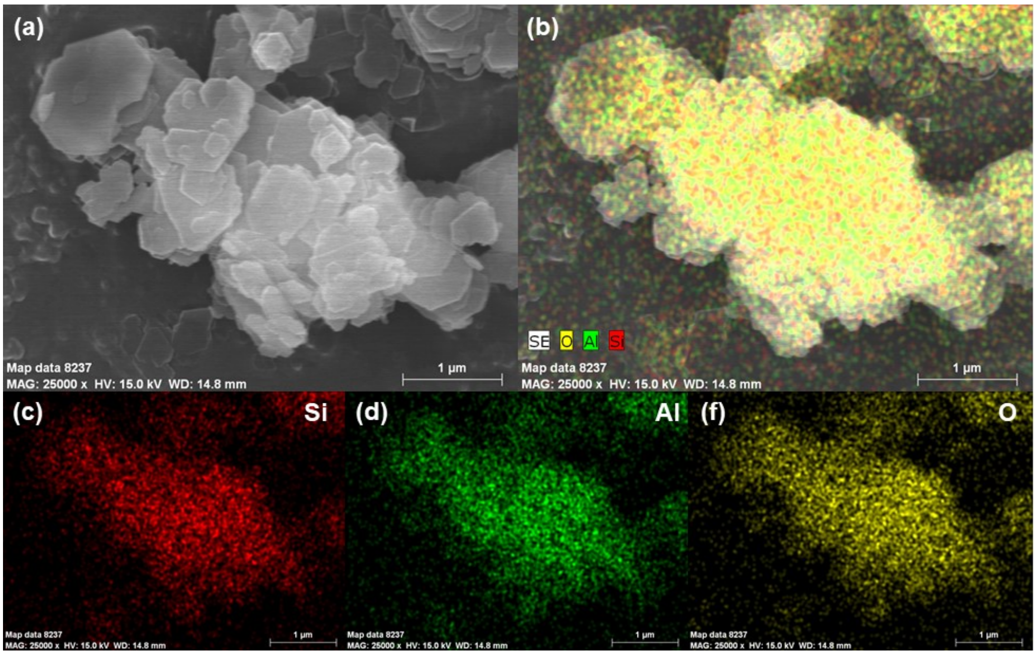

Fig. S2. The Energy Dispersive Spectrometry images (EDS) of KC

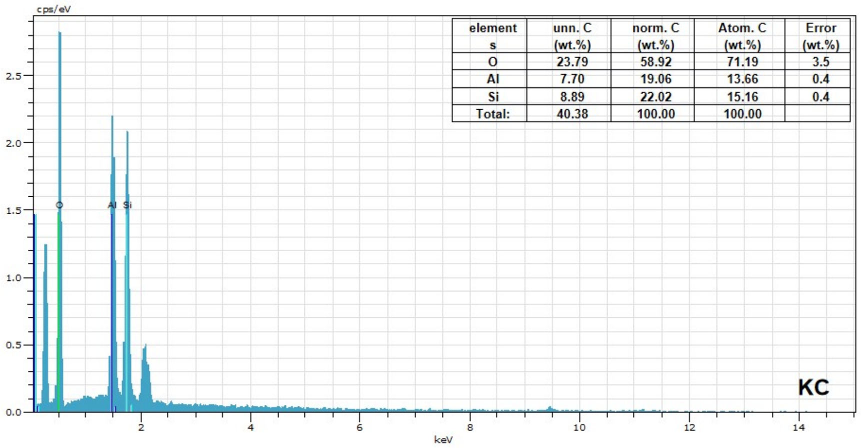

Fig. S3. The surface elements content of KC.

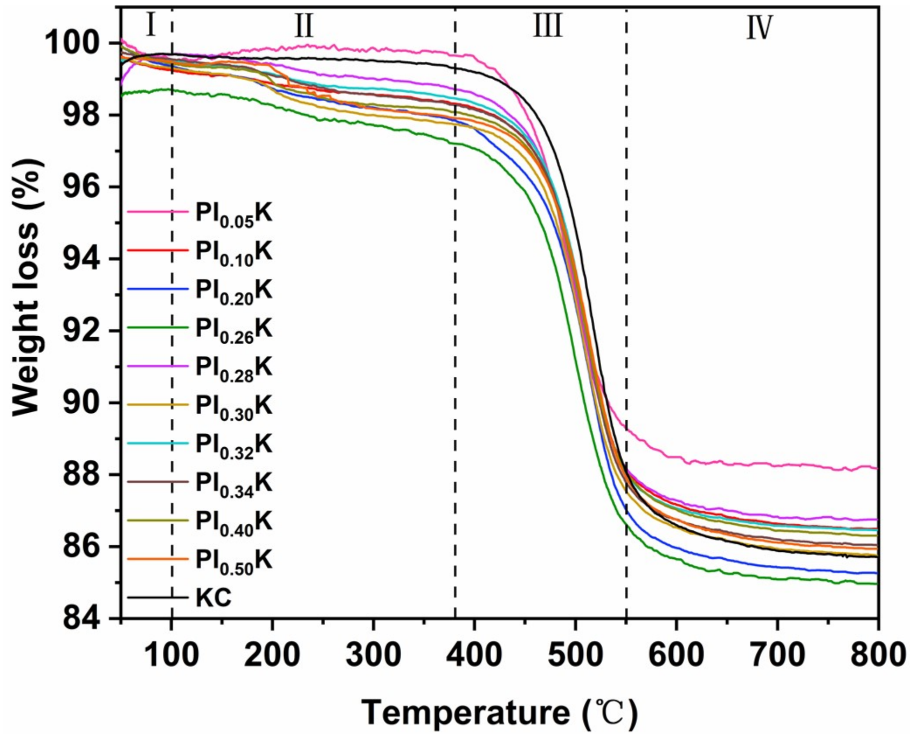

Fig. S4. The relationship between sample temperature variation and weight loss rate.

Table S2. Thermogravimetric loss of KC and loading ratio of PI to the surface of modified kaolin.

Materials	Thermogravimetric loss ratio (%)	Residual mass ratio (%)
KC	11.93	88.07
$PI_{0.05}K$	10.71	89.29
$PI_{0.10}K$	11.84	88.16
$PI_{0.20}K$	12.98	87.02
$PI_{0.26}K$	13.38	86.62
$PI_{0.28}K$	11.82	88.18
$PI_{0.30}K$	12.46	87.54
$PI_{0.32}K$	11.97	88.03
$PI_{0.34}K$	12.23	87.77
$PI_{0.40}K$	11.93	88.07
$PI_{0.50}K$	12.08	87.92

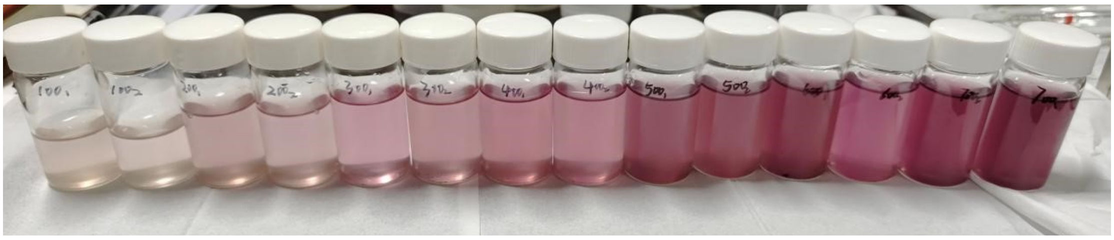

Fig. S5: Comparison of the Effect of PIK Adsorption on Direct Black 38 Dyes with Different Low Concentrations (100-700 mg·L^{-1})

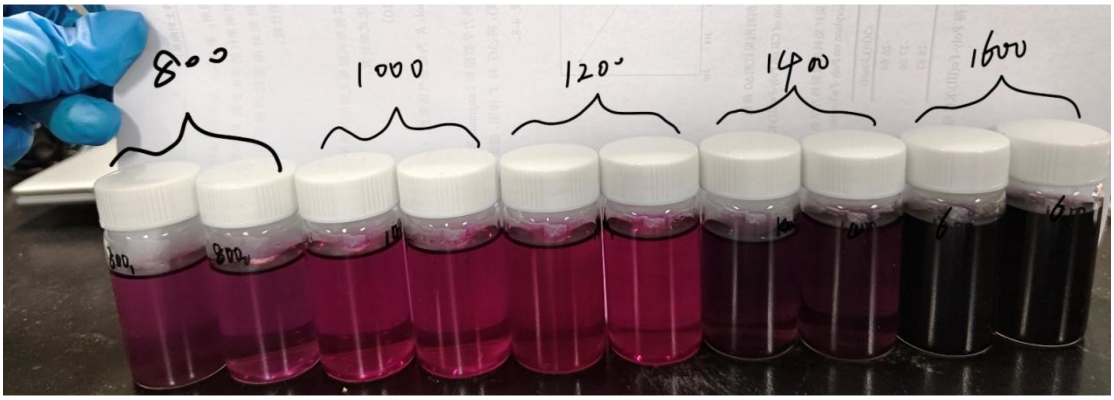

Fig. S6: Comparison of the effects of PIK adsorption of different high concentrations (800-1600 mg·L^{-1}) of DB 38 dye

Table. S3 Changes in solution pH before and after adsorption

Before	3.00	4.00	5.00	6.00	7.00	8.00	9.00	10.00	11.00	12.00
After	2.54	3.67	3.69	4.77	5.82	5.88	6.02	7.54	7.70	8.45

Table S4. The adsorption capacity of DB38 on different adsorbents not similar and compatible with PIK.

Adsorbent		Adsorption conditions				Max.adsorption capacity	Reference
Material	Modification method	Dosage ($g\ L^{-1}$)	Concentration ($mg\ L^{-1}$)	Time (min)	Temperature (°C)	($mg\ g^{-1}$)	
Struvite	Precipitation method	10.0	80-200	80	20	38.14	Foletto EL et al.[8]
Yerba mate	$ZnCl_2$	0.5	70-300	50	25	244.5	Linhares B et al.[9]
Coal tar	Zinc acetate Template method coupled with KOH activation	/	50-700	1440	25	294	I.I. Shahib et al.[10]
In-TATAB（MOF）	MBB method	1	50-600	1440	room temperature	259	N.Chaukura et al.[11]
E. faecalis R1107	/	/	50-1000	2880	37		A.Chaoui et al.[12]
p-Diethanolaminomethylcalix[4]arene.	Organic synthesis	6	15	45	25	26696	Junejo R et al.[13]
Bottle gourd	KOH active	0.5	70-200	60	25	94.9	Foletto EL et al.[14]
MCM-41	Sol-gel method	2	20-600	120	25	79.9	Tugce A. Arica et al.[15]
Peanut shell	Microwave irradiation followed by pyrolysis	2	100-350	40	25	110.6	Georgin J et al.[16]
Petroleum pitch	Synthesis of hierarchical porous carbon and Zn(II) modification	1	20-200	1440	25	438.6	Zhang C et al.[17]
Zeolites	alkaline fusion coupled with hydrothermal treatment	5	50-500	40	25	13.64	Rafique M et al.[18]
Kaolin clay	Polymer hydroxylated iron modification	10	100-1600	90	25	76.9	This work

Table S5 The adsorption performance of different iron modified materials on pollutants

Adsorbent			Adsorption conditions				Maximum adsorption capacity (mg g^{-1})	
Raw materials	Modification method	Pollutants	Dosage (g L^{-1})	Concentration (mg L^{-1})	Time (min)	Temperature (°C)		Reference
Montmorillonite	Fe^{3+} Polyhydroxy cations	Phosphate	10	10-100	180	25	2.50	Leite ST et al.[19]
Bentonite	Fe^{3+} Impregnation and precipitation	Rhodamine B	0.15	10-50	90	25	98.62	Hou MF et al.[20]
Clinoptilolite	Polymeric iron/zirconium	Phosphate	2.5	2	90	25	13.76	Zhou K et al.[21]
Coal-bearing kaolin	High temperature calcination and acid treatment	Fluorine	10	2.5-50	180	25	11.09	Muschin T et al.[22]
Coal-bearing kaolin	Ball milling and impregnation polymerization	Congo red	10	200-1200	180	25	75	Muschin T et al.[23]
Vermiculite	Fe^{3+} Polyhydroxy Cations	Phosphate	10	3.10-21.70	200	25	16.9	Do Nascimento FH et al.[24]
Montmorillonite	Hydroxyl iron pillared and cationic panthenolintercalation	Benzothiazoles	0.5	20-140	90	25	74.35	Hua Qiang et al.[25]
Kaolin clay	Polymerhydroxylated iron modification	Direct black 38	10	100-1600	90	25	76.9	This work

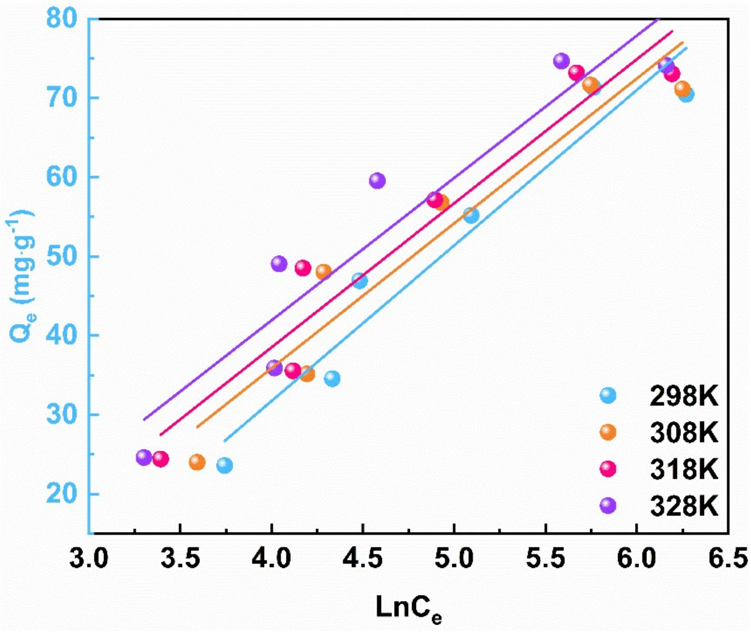

Fig. S7. Temkin isotherm adsorption fitting curves of PIK adsorption on DB38.

Table. S6. Results obtained from fitting Temkin models for DB38 sorption by PIK.

T/*K*	Temkin isotherm		
	A_T/(L·g^{-1})	b_T/(J·mol^{-1})	R^2
298	9.281	126.21	0.9354
308	7.652	140.08	0.9189
318	6.554	145.43	0.9136
328	5.283	151.75	0.8956

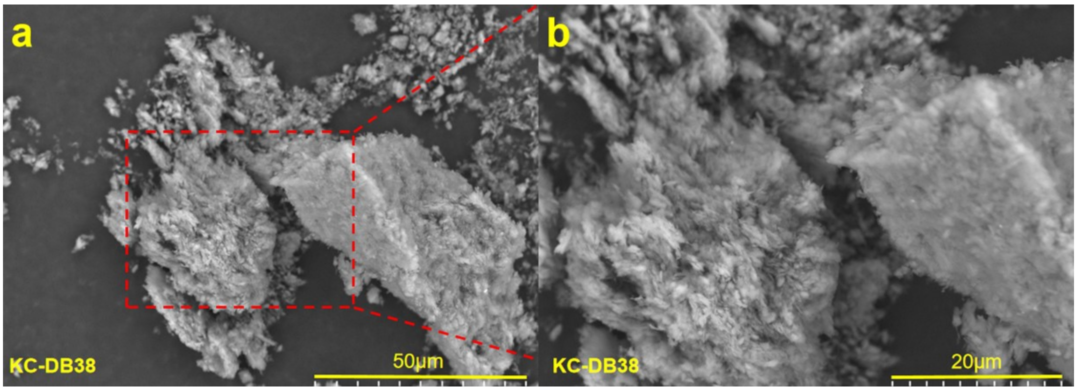

Fig. S8. SEM image of KC-DB38.

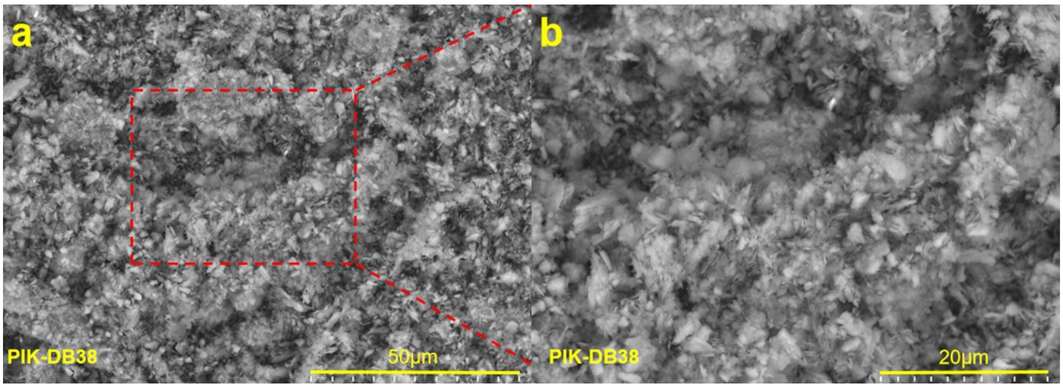

Fig. S9. SEM images and EDS of PIK after adsorption of DB38.

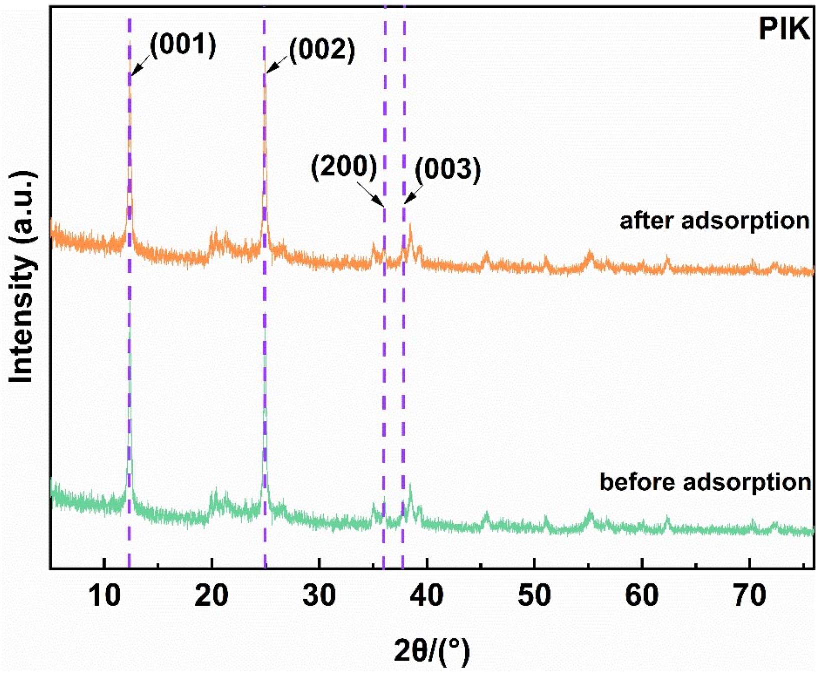

Fig. S10. XRD images of PIK before and after adsorption of DB38.

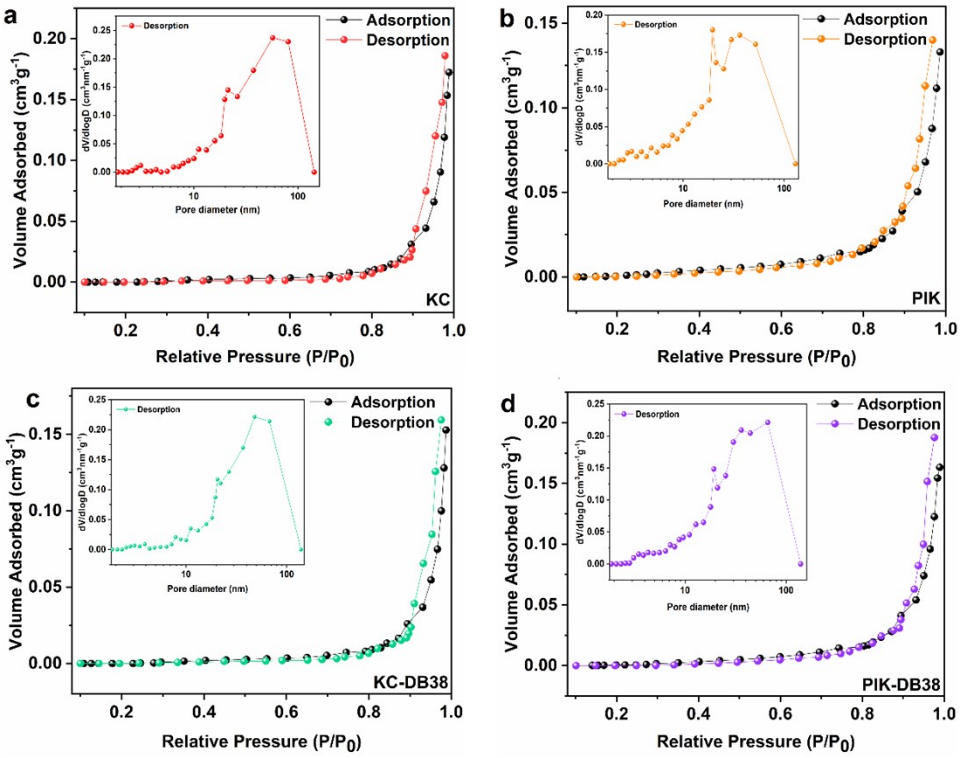

Fig. S11. The N_2 adsorption-desorption isotherms and pore size distribution (a. KC, b. PIK, c. KC-DB38, d. PIK-DB38).

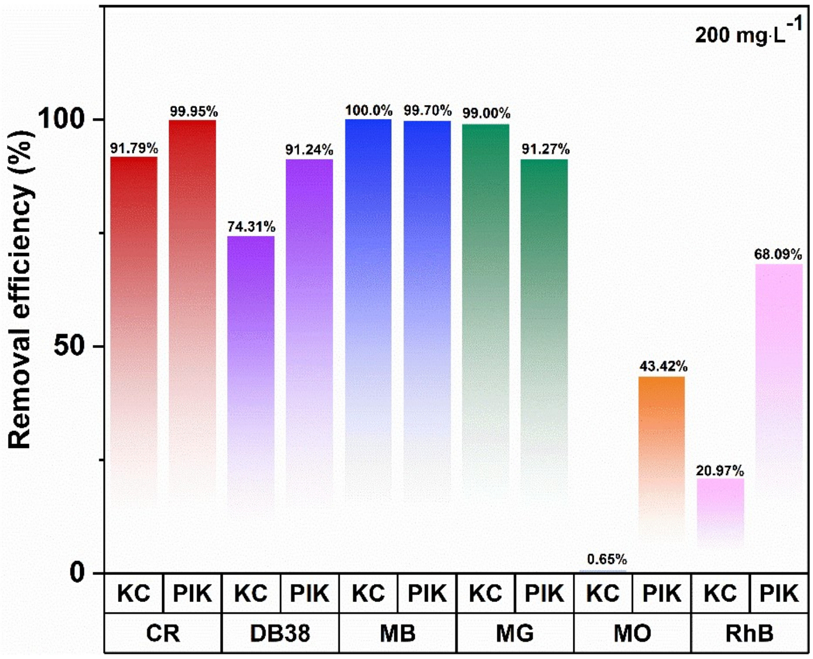

Fig. S12: Removal efficiency of Congo Red (CR), Direct Black 38 (DB38), Methylene Blue (MB), Malachite Green (MG), Rhodamine B (RhB), Methyl Orange (MO) by Kaolin Clay (KC) and Polymer hydroxylated iron modified kaolin ($PI_{0.26}K$) in solution.

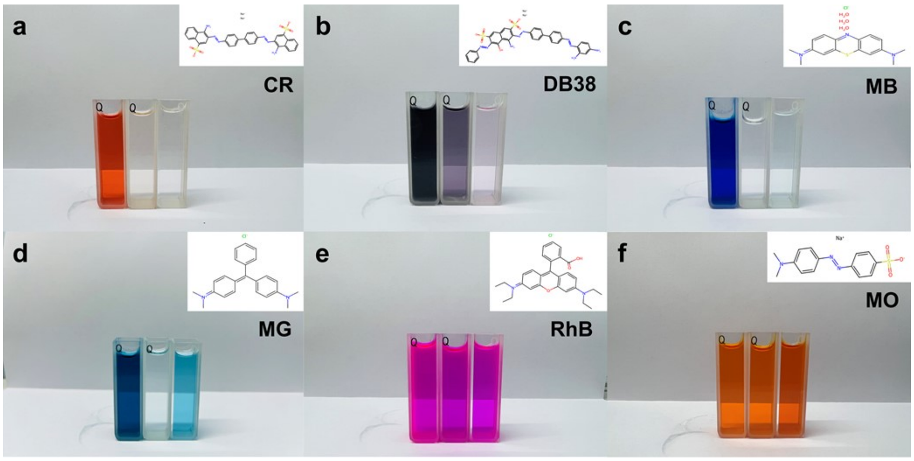

Fig. S13: Comparison of PIK adsorption effects on different types of dyes (a. Congo Red, b. Direct Black 38, c. Methylene Blue, d. Malachite Green, e. Rhodamine B, f. Methyl Orange), Each group consists of 200 mg·L^{-1} original solution, KC adsorbed dye, and PIK adsorbed dye.

The adsorption effect of PIK on low concentration azo dyes CR, DB38, MO, and triphenylmethane dyes RhB is superior to that of KC (Fig. S12 and S13). CR, DB38,

and MO are all anionic dyes, and their molecular structures contain functional groups such as –OH, $–NH_2$, which facilitate PIK adsorption. Although RhB is a cationic dye, it can also be adsorbed by PIK because its molecular structure also contains –COOH, which can form hydrogen bonds with the functional groups on the surface of the adsorbent PIK. However, the adsorption efficiency of PIK on thiazide dye MB and triphenylmethane dye MG is significantly lower than that of KC. These two dyes are cationic dyes and have no functional groups in their own structures that can form hydrogen bonds with PIK. Due to the negative charge of PIK, it exhibits strong electrostatic repulsion with MB and MG in pH neutral solutions, making adsorption unfavorable. In conclusion, PIK exhibits good adsorption capacity for various dye pollutants. But in practical applications, the characteristics of pollutants and environmental conditions should be taken into account to maximize adsorption capacity of the adsorbent.

Table S7. XPS valence state analysis and content of KC, PIK, and PIK-DB38.

Name	KC		PIK		PIK-DB38	
	Peak BE/eV	Atomic %	Peak BE/eV	Atomic %	Peak BE/eV	Atomic %
Al2p Al^{3+}	74.69	10.33	74.73	10.2	75.1	11.26
C1s C–C	284.8	13.63	284.8	9.72	284.8	6.87
C1s C–O	286.46	2.07	286.38	2.14	286.29	1.92
C1s C=O	288.85	1.11	288.76	0.86	288.67	1.17
Fe2p3 Fe^{2+}	710.3	0.11	710.34	0.87	710.55	0.576
Fe2p3 Fe^{3+}	712.4	0.09	712.21	0.78	712.38	0.59
K2p3 K^{+}	293.24	0.12	293.42	0.4	293.48	0.2
O1s Al-O	531.88	42.4	531.96	40.43	531.99	29.67
O1s Fe-O	530.05	1.3	530	3.62	530.15	3.33
O1s OH	534.35	2.47	534.48	2.38	534.53	2.92
O1s Si-O	532.68	12.61	532.76	15.17	532.79	26.18
Si2p SiO_2	102.96	13.77	103.09	13.42	103.31	13.78
N1s C-N	–	–	–	–	399.79	0.84
N1s N-O	–	–	–	–	402.06	0.17
S2p Sulfate	–	–	–	–	168.42	0.33

Table S8. The leaching amount of Fe ions from different adsorbent.

Samples	First cycle Fe Leaching amount ($ug \cdot L^{-1}$)	Second cycle Fe Leaching amount ($ug \cdot L^{-1}$)	Third cycle Fe Leaching amount ($ug \cdot L^{-1}$)
PIK	34.39	48.71	105.10
Fe-KC	214.02	156.76	158.18

Table S9. Molecular Structures and Key Physicochemical Properties of the Six Dyes: Solubility, pK_a, and logK_{ow}.

Dyes	Molecular Structure	Solubility (298K)	p_{Ka} (298K)	logk_{OW}
Methyl Orange	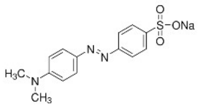	5 g/L	3.4	4.15290
Congo Red	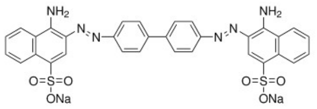	10 mg/mL	4.1	10.78740
Direct Black 38	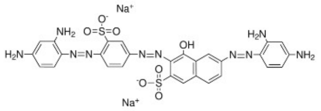	5.00 mg/mL	/	11.91860
Rhodamine B	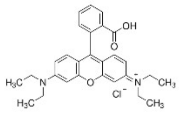	100 mg/mL	3.7~4.32	2.56500
Malachite Green	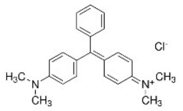	5.00 mg/mL	13.78±0.29	1.39760
Methylene Blue	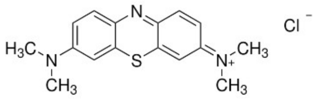	40 g/L	2.6, 11.2	-0.4971

References

1. T. Muschin, H. Zulchin and M. Jia, *ChemistrySelect*, 2021, **6**, 3075-3083.
2. X.-J. Liu, M.-F. Li and S. K. Singh, *Journal of Materials Research and Technology*, 2021, **12**, 1434-1445.
3. Y. Sun, T. Wang, C. Han, X. Lv, L. Bai, X. Sun and P. Zhang, *Bioresource Technology*, 2022, **344**, 126186.
4. X. Su, X. Wang, Z. Ge, Z. Bao, L. Lin, Y. Chen, W. Dai, Y. Sun, H. Yuan and W. Yang, *Chemical Engineering Journal*, 2024, **486**, 150387.
5. B. Linhares, C. T. Weber, E. L. Foletto, D. S. Paz, M. A. Mazutti and G. C. Collazzo, *Environmental technology*, 2013, **34**, 2401-2406.
6. E. L. Foletto, C. T. Weber, D. S. Paz, M. A. Mazutti, L. Meili, M. M. Bassaco and G. C. Collazzo, *Water science and technology*, 2013, **67**, 201-209.
7. T. A. Arica, E. Ayas and M. Y. Arica, *Microporous and Mesoporous Materials*, 2017, **243**, 164-175.
8. F. EL, B. S, M. MA and J. SL., *Chemical Engineering Communications*, 2013, **8**, 12.
9. L. B, W. CT, F. EL, P. DS, M. MA and C. GC, *Environmental technology*, 2013, **16**, 6.
10. Z. C, Z. W, Y. M, N. K, Z. L, W. X and H. X, *Journal of Porous Materials*, 2017, DOI: 10.1007/s10934-017-0369-2, 4.
11. L. X, L. B, E. JF and L. Y, *Materials Chemistry Frontiers.*, 2020, **1**, 7.
12. A. Chaoui, S. Farsad, A. B. Hamou, A. Amjlef, N. Nouj, M. Ezzahery and N. E. Alem, *Journal of Environmental Management*, 2024, **351**.
13. J. R, M. S and K. S, *Journal of Chemical & Engineering Data*, 2020, **10**, 10.
14. F. EL, W. CT, P. DS, M. MA, M. L, B. MM and C. GC, *Water science and technology*, 2013, **1**, 10.
15. A. TA, A. E and A. MY, *Microporous and Mesoporous Materials*, 2017, **243**, 12.
16. G. J, D. GL, M. MA and F. EL, *Journal of Environmental Chemical Engineering*, 2016, **1**, 10.
17. Z. C, L. Y, L. Y, Z. W, W. X, H. X and Y. M, *Chemosphere*, 2019, **216**, 7.
18. R. M, C. SA, A. A, S. S and H. T, *Kuwait Journal of Science*, 2023, **4**, 7.
19. S. T. Leite, F. H. d. Nascimento and J. C. Masini, *Heliyon*, 2020, **6**.
20. H. MF, M. CX, Z. WD, T. XY, F. YN and W. HF, *Journal of Hazardous Materials*, 2011, **186**, 6.
21. Z. K, W. B, D. X and C. X, *Chemical Engineering Journal*, 2018, **347**, 9.
22. M. T, Z. H and J. M, *ChemistrySelect*, 2021, **13**, 9.
23. M. T, D. X, B. U, Z. H and A. B, *ChemistrySelect*, 2019, **47**, 7.
24. d. N. FH and M. JC, *Minerals*, 2022, **8**, 9.
25. Q. H, W. F, X. M, L. W and W. X, *Colloids and Surfaces A: Physicochemical and Engineering Aspects*, 2021, **626**.
